# Self-management support using an Internet-linked tablet computer (the EDGE platform)-based intervention in chronic obstructive pulmonary disease: protocol for the EDGE-COPD randomised controlled trial

**DOI:** 10.1136/bmjopen-2013-004437

**Published:** 2014-01-08

**Authors:** Andrew Farmer, Christy Toms, Maxine Hardinge, Veronika Williams, Heather Rutter, Lionel Tarassenko

**Affiliations:** 1 Department of Primary Care Health Sciences, University of Oxford, Oxford, UK; 2 Oxford University Hospitals NHS Trust, Oxford, UK; 3 Oxford Health NHS Foundation Trust, Oxford, UK; 4 Institute of Biomedical Engineering, University of Oxford, Oxford, UK

**Keywords:** PRIMARY CARE, RESPIRATORY MEDICINE (see Thoracic Medicine)

## Abstract

**Introduction:**

The potential for telehealth-based interventions to provide remote support, education and improve self-management for long-term conditions is increasingly recognised. This trial aims to determine whether an intervention delivered through an easy-to-use tablet computer can improve the quality of life of patients with chronic obstructive pulmonary disease (COPD) by providing personalised self-management information and education.

**Methods and analysis:**

The EDGE (sElf management anD support proGrammE) for COPD is a multicentre, randomised controlled trial designed to assess the efficacy of an Internet-linked tablet computer-based intervention (the EDGE platform) in improving quality of life in patients with moderate to very severe COPD compared with usual care. Eligible patients are randomly allocated to receive the tablet computer-based intervention or usual care in a 2:1 ratio using a web-based randomisation system. Participants are recruited from respiratory outpatient clinics and pulmonary rehabilitation courses as well as from those recently discharged from hospital with a COPD-related admission and from primary care clinics. Participants allocated to the tablet computer-based intervention complete a daily symptom diary and record clinical symptoms using a Bluetooth-linked pulse oximeter. Participants allocated to receive usual care are provided with all the information given to those allocated to the intervention but without the use of the tablet computer or the facility to monitor their symptoms or physiological variables. The primary outcome of quality of life is measured using the St George's Respiratory Questionnaire for COPD patients (SGRQ-C) baseline, 6 and 12 months. Secondary outcome measures are recorded at these intervals in addition to 3 months.

**Ethics and dissemination:**

The Research Ethics Committee for Berkshire—South Central has provided ethical approval for the conduct of the study in the recruiting regions. The results of the study will be disseminated through peer review publications and conference presentations.

**Trial registration:**

Current controlled trials ISRCTN40367841.

**Strengths and limitations of this study**

- The study focusses on the impact of a self-management intervention delivered through a low-cost Internet enabled tablet computer.
- The study is powered to examine quality of life outcomes and uses an unbalanced allocation to examine the effect of the EDGE (sElf management anD support programme) platform across a wide range of participants.
- The study is not sufficiently large enough to provide a detailed cost-effectiveness evaluation, or to provide sufficient power to demonstrate clinically important differences between intervention and usual care groups for hospital admission rates.

## Background

Chronic obstructive pulmonary disease (COPD) is an important cause of morbidity and mortality worldwide responsible for three million deaths globally per year.^1^ In the UK, the total annual estimated cost of COPD to the National Health Service (NHS) is over £800 million, with over half of this attributable to hospital-based care,^2^ and the impact of COPD to the health-related quality of life of patients is well established.^3^ ^4^ There is now promising evidence that training and support for patients in the self-management of their condition improves quality of life and can reduce unplanned hospital admissions.^5^ ^6^ However, the results of individual studies are mixed,^7^ and the challenge remains to identify those for whom different forms of self-management are suitable and to develop and optimise ways of delivering available interventions to maximise effectiveness and safety.

Use of the converging computer and communication technologies in the form of telehealth-based interventions offers a means of helping patients monitor their condition, providing support in interpreting data, providing a means of delivering individually tailored education and treatment plans and allowing clinicians to monitor long-term trends and identify short-term safety issues.

Evaluation of telehealth-based interventions can be complex with a requirement for considering multiple perspectives within the evaluation.^8^ For a successful evaluation, there is a need to improve the delivery of clinical care, control the workload placed on healthcare professionals and to provide cost-effective improvements in treatment outcomes.

Systematic reviews of telehealth in COPD provide evidence to support continuing research,^9^ but recent large-scale evaluations of telehealth-based treatment programmes have not shown convincing evidence of effectiveness.^10^ ^11^ The challenge remains to ensure that the telehealth systems can be easily used by patients and that the systems integrated into the healthcare system are acceptable to clinicians and patients. Criticism of previous systems has included difficulty with data entry, leading to lifestyle restriction, unreliability of monitoring devices and lack of integration within an individual patient's day-to-day life.

Our group has long experience of developing and testing healthcare interventions based on the use of mobile phones and their integration into clinical care delivery. Mobile phones or tablet computers with a subscriber identity module (SIM) card provide a platform in which communication and computing technologies are sustainably integrated and a platform for communicating with monitoring devices. We have recently carried out a cohort study in which we have shown that a tablet-computer based system for supporting patients with COPD is acceptable and feasible.^12^

We have therefore set out to determine the efficacy of an Internet-linked tablet computer-based intervention (the EDGE—sElf management anD support proGrammE—platform), with patients with moderate to very severe COPD, in improving quality of life measured with the St Georges Respiratory Questionnaire for COPD patients (SGRQ-C) in comparison with standardised usual care. In addition, we will collect data on morbidity, mortality and hospital admissions to inform the design of future evaluations of the system.

## Methods

### Trial design

**Figure 1:**
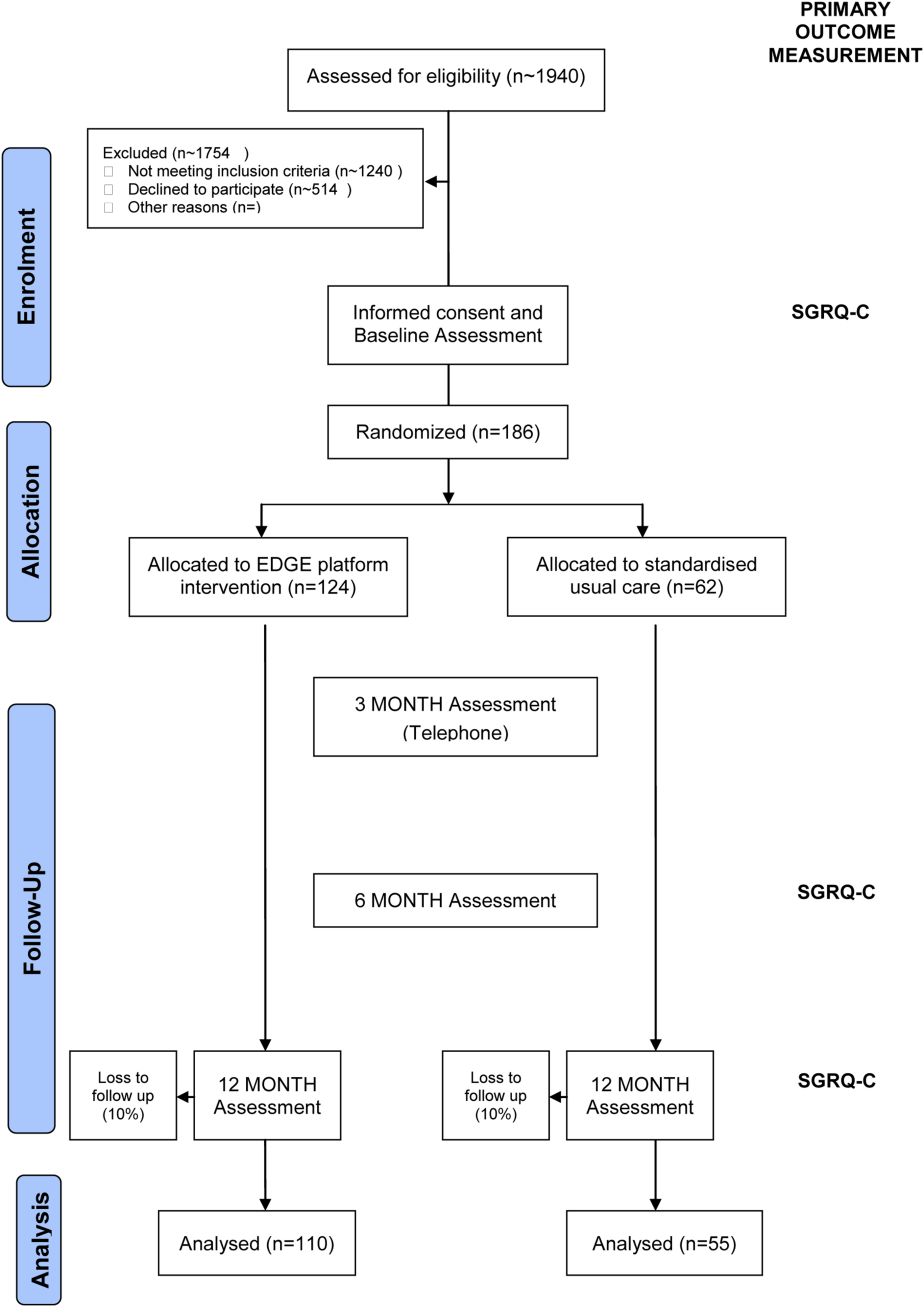
Patient flow. EDGE, sElf management anD support programme; SGRQ-C, St George's Respiratory Questionnaire for COPD patients.

The EDGE for COPD is a multicentre, randomised controlled trial of 12-month duration. Patients are individually randomised to receive either an Internet-linked tablet computer (the EDGE platform)-based intervention or standardised usual care in a 2:1 allocation ratio (figure 1).

### Participants

#### Eligibility criteria for participants

Eligible patients are those aged ≥40 years with a confirmed diagnosis of COPD defined as a forced expiratory volume in one second (FEV_1_), post-bronchodilation of <80% and a predicted ratio of FEV_1_ to forced vital capacity of <0.70. Eligible patients have a smoking-pack history >10 pack-years and a Medical Research Council dyspnoea score ≥2. A clinical decision of trial suitability for patients who are unable, for clinical reasons, to provide a spirometry reading at full assessment is sufficient for eligibility if the patient has prior clinical evidence of COPD (eg, obstructive spirometry within the past 10 years or radiological evidence of emphysema). Patients are required to be registered with a general practitioner and have had an exacerbation of COPD requiring home treatment or hospital admission in the previous year or have been referred for pulmonary rehabilitation.

Patients are not eligible for trial participation if they have other significant lung disease or chronic heart failure (defined by the New York Heart Association classification system as severe (grade IV)) or a life expectancy of <3 months. Eligible patients must be able to provide informed consent, to complete the trial questionnaires and not be cognitively impaired. Patients living in areas without access to an Internet-enabled mobile phone network and thus unable to transmit and receive data are not eligible to enter the trial.

#### Setting

To maximise recruitment of participants to the trial, patients are identified from a variety of settings encompassing primary and secondary care as well as community services. Patients attending respiratory hospital outpatient clinics and pulmonary rehabilitation courses in the adjacent counties of Oxfordshire and Berkshire, UK, are invited to participate. In addition, eligible patients are identified from primary care clinics and from those recently (within the preceding 2 weeks) discharged from hospital following a COPD-related admission.

### Trial interventions

#### Intervention development and specification

We developed and tested an intervention to support patients with COPD in monitoring their health and to provide information and education about their condition based on use of an Internet-linked tablet computer (the EDGE platform). Based on open architecture application software, the EDGE platform was developed to allow integration within clinical care by a team of clinicians and engineers working with patients. The platform was refined in a 6-month cohort study with a group of patients with COPD selected using eligibility criteria matching those of the trial. Key principles underlying the development of the platform include ease of use for a group of participants less experienced with computers (large icons, no keyboard needed for data entry) and ensuring data quality (tablet-based instructions to ensure ease of use, development of algorithms to assure data quality).

The EDGE platform intervention incorporates a daily symptom diary consisting of a series of standard questions about symptoms based on previous trial protocols.^2^ ^13^ ^14^ Questions include general well-being, cough, breathlessness, sputum (quantity produced and colour) and use of medications. A 30 second period of data transmission using a Bluetooth-enabled pulse oximeter with finger probe allows daily collection of pulse rate and oxygen saturation data. Every 4 weeks, beginning 2 weeks after initial use, the platform presents the four-item Patient Health Questionnaire (PHQ-4) screening measure^5^ ^15^ and if either the depression or anxiety components score is ≥3 then the relevant full questionnaire (depression (PHQ-8)^6^ ^16^) or Generalised Anxiety Disorder 7-item (GAD-7^7^ ^17^), is presented for completion.

The EDGE platform also includes software modules to provide patients with additional support for self-management of their condition. These include (1) personalised plans for self-management and treating an exacerbation of their condition; (2) brief video clips and text-based material providing additional information about COPD and treatments (including medicines use and inhaler technique), and educational advice on managing COPD, smoking cessation, diet, physical activity and mood management (depression and anxiety); and (3) the facility to receive a brief message from their respiratory nurse. Prompts for accessing the physical activity and mood management information are included for patients reporting higher scores on the depression and anxiety screening measures.

The data entered onto the EDGE platform is transmitted immediately to a server hosted behind NHS firewalls. The data held on the server is reviewed at no less than 4-day intervals by a clinician to identify technical problems and review clinical data received.

In an initial 6-week period of use, EDGE-platform users complete the symptom diary and record their oxygen saturation and heart rate with the pulse oximeter on a daily basis. Following this initial run-in period of use, the distributions of values for the oxygen saturation, heart rate and symptom scores are calculated for the run-in period for each participant. The 95th centile is computed for each distribution and used as the threshold for the participant's safety alert for that parameter. Every time the participant-specific threshold for a parameter is crossed after the run-in period, the safety alert is displayed on the web-based record for that participant.

#### The EDGE platform-based intervention

Participants allocated to receive the Internet-linked tablet computer (EDGE platform)-based intervention are provided with an Android tablet computer (Samsung Galaxy Tab) running the application software and Bluetooth-enabled oximeter probe (Nonin, PureSAT, 956OBT, Nonin Medical Inc, Plymouth, Minnesota, USA). Participants are briefly instructed on the use of the EDGE platform by the research nurse and are provided with a brief information booklet giving details of its use. Participants continue to input their symptom data and clinical recordings daily throughout the duration of the trial. Patients are informed that the EDGE system is not a replacement for their usual clinical care, and that in the event of deterioration in their health they should contact their general practitioner or community respiratory nurse as usual. However, data are reviewed by a clinician at no less than 4-day intervals, and changes in heart rate, oxygen saturation or symptom score that cross the participant's specific threshold for that parameter and persist for more than 4 days are followed up with a phone call to either the patient, general practitioner or community respiratory nurse as appropriate. If depression or anxiety scores equal or exceed a threshold of 10, then the general practitioner is informed by letter.

#### The standardised usual care intervention

Participants allocated to receive standardised usual care are provided with all the information given to those allocated to use the EDGE platform, but without the use of a tablet computer or the facility for daily monitoring of symptoms and physiological variables. The research nurse provides participants with leaflets based on those currently produced by the Oxfordshire Community Respiratory service. Personalised information intended to help patients understand their condition includes information about how to use their medications and when they should be used, a self-management plan with written guidelines on what to do and whom to contact if they experience an exacerbation and dietary advice is provided. If the participant has not attended a pulmonary rehabilitation course they are invited to do so.

### Outcomes

Trial outcome measures will be recorded as indicated in table 1.

**Table 1:** Trial outcome measures

	Baseline assessment visit	3 months	6 months	12 months
Primary outcome				
SGRQ-C (quality of life)^18^	x		x	x
Secondary outcomes				
Number of exacerbations	x	x	x	x
Time to first exacerbation (days)		x	x	x
Unplanned healthcare contacts		x	x	x
EuroQol 5 Dimension Questionnaire^22^	x		x	x
Self-completed 20 item depression measure D^20^	x		x	x
Self-completed 10 item anxiety measure^21^	x		x	x
Beliefs about Medicines Questionnaire^19^	x		x	x
Medication adherence report schedule^19^	x		x	x
Prescribed medicines	x	x	x	x
Smoking status	x		x	x
Health service usage	x	x	x	x
Death		x	x	x
Admission to hospital	x	x	x	x
Days out hospital	x	x	x	x

SGRQ-C, St George's Respiratory Questionnaire for COPD patients.

#### Primary outcome measure

The primary outcome is the SGRQ-C^8^ ^18^ used to assess quality of life in patients with moderate to severe COPD.

#### Secondary outcome measures

The following secondary endpoints will be used to evaluate the impact of the intervention in comparison with usual care (1) impact on hospital admissions (number of admissions and days out of hospital) and deaths; (2) the number of recorded exacerbations defined as episodes in which antibiotics or oral steroids were prescribed or in which the patients were seen in the accident and emergency department and/or admitted to hospital as a result of a respiratory episode; (3) time to first exacerbation; (4) beliefs about respiratory medicine use measured with the Beliefs about Medicines Questionnaire;^9^ ^19^ (5) self-reported medication use measured with the Medication Adherence Report Schedule;^10^ ^11^ ^19^ (6) self-reported smoking cessation; (7) mood measured with the Standard Checklist 20-item Questionnaire (SCL-20) for depression^20^ and the Standard Checklist 10-item Anxiety Measure (SCL-10A)^21^ and (8) health status measured with the EuroQol 5-Dimension Questionnaire (EQ-5D).^22^

Details of number and duration of hospital admissions will be measured by self-report and confirmed where possible by a review of hospital discharge letters and central hospital admissions data. Records of deaths will be obtained from general practices and further details will be obtained, where necessary, from hospital records.

Details of exacerbations of COPD will be recorded on a record form held by all participants. This will include number, severity, medications prescribed and outcomes to enable, where possible, details to be confirmed by review of medical records or, if deterioration results in admission, review of hospital records.

Costs of healthcare will be identified using a brief questionnaire to obtain self-reported information about visits to general practitioners. Standard costs will be used for hospital admissions and prescribed respiratory medicine.

### Sample size

The sample size calculations are based on the number of patients required to demonstrate a mean difference of 6.6 on the St George's Respiratory Questionnaire between the two trial groups to which participants are allocated, over a 12-month period (equivalent to 7.3 on SGRQ-C).^18^ Although there is limited data on this outcome in settings using computer-based interventions, we have estimated the SD at 12.7 based on a study using the SGRQ-C.^23^ A trial using these estimates with a power of 90% and significance level of 0.05 (2-sided), with 2:1 allocation between intervention and usual care and allowing for 10% loss to follow-up would require 165 patients.

We also have 98% power to identify the difference in admissions to hospital at 3 months based on effect sizes of previous intensive interventions with this group of patients^24^ and 52% power to detect the difference in admissions at 12 months based on a systematic review of interventions in COPD.^25^ In both cases, a 5% loss to follow-up has been assumed.

### Randomisation

Participants are randomised with an allocation ratio of 2:1 intervention to usual care using Sortition V.1.2 (an online, web-based randomisation system developed by the Primary Care Clinical Trials Unit at the University of Oxford). A computer schedule based on recruiting site (Oxfordshire or Berkshire), age (≤70 years or >70 years), gender, COPD severity (moderate or severe/very severe) and current smoking status (yes or no) is used to minimise imbalance between the groups,^26^ and is monitored by an independent statistician. The research nurse randomises the patient by accessing Sortition using a web-browser on a tablet computer at the assessment visit only after completion of consent procedures and baseline measurements, including completion of the SGRQ-C.

Self-completed outcome measures at 6 and 12 months are completed without guidance by the research team and prior to any further assessment or discussion of clinical care. Research and clinical teams are trained in the potential for measures to be biased by their interactions with participants. A record of all contacts with trial participants is kept to examine potential for interactions with patients not specified in the trial protocol.

### Trial procedures

#### Recruitment

Potentially eligible patients are identified from those discharged from hospital following a COPD-related admission, from respiratory hospital outpatient clinics, pulmonary rehabilitation courses and from primary care clinics, and are sent an invitation to participate in the trial. The invitation includes a patient information booklet, a reply slip and prepaid envelope. Patients interested in participating are asked to return their reply slips by post to the research team. The research nurse then contacts the patient by telephone to arrange an initial assessment visit. At this visit eligibility is confirmed, written informed consent is obtained and baseline data are collected for those consenting to participate.

All participants are assessed at baseline by a healthcare professional and complete self-completed measures prior to randomisation and intervention allocation. The use of medication by participants is recorded at the baseline and follow-up assessment visits. Information collected includes type, dose and frequency of COPD medication (tablets and inhalers) as well as a list of other medication taken. A detailed smoking history is taken at the baseline assessment visit; self-reported smoking status is recorded at subsequent assessments.

#### Patient follow-up and retention

Patients remain in the trial for 12 months with assessments at a baseline visit, 3, 6 and 12 months. The primary outcome measure is collected at baseline, and 6 and 12 months after randomisation. Secondary outcome measures are collected at baseline, 3, 6 and 12 months.

The 3-month assessment is a telephone contact with patients sent reminders prior to the assessment date. For patients allocated to standardised usual care, a reminder is posted prior to the assessment date. For patients receiving the EDGE platform intervention, this reminder is sent in the form of a message to the tablet computer. The 6 and 12 months visits are carried out either at home or at clinic, with patients receiving postal or electronic reminders prior to the assessment visit date. The researcher ensures at each visit that all outcome measures are complete to ensure maximal follow-up data are collected.

All patients have the right to withdraw from the trial at any point, without providing a reason. Those patients who do withdraw from the trial will be asked if they would be willing to provide follow-up information at the 6 and 12 months assessment points. If patients decline, no further information will be collected.

### Statistical methods

The principal comparisons will be performed on an intention-to-treat basis. The trial results will be presented as comparative summary statistics (difference in response rates or means) with 95% CI. If appropriate, and depending on the distribution of the continuous outcome measures, a linear mixed-effects model will be used to analyse SGRQ-C over the 12-month period of the trial, adjusting for baseline value and minimisation covariates. Treatment-time interaction will be included in the model to assess the treatment effect at 12 months. We will formally assess the distribution of the change from baseline for evidence of departure from normality. If necessary, data will be either transformed or analysed using a non-parametric equivalent. The nature and mechanism for the missing outcomes will be investigated, though mixed-effects model implicitly account for data missing at random. Sensitivity analyses will be carried out to examine the robustness of the results with different assumptions about departures from randomisation policies, and handling of missing data. Binary outcomes will be analysed using log-binomial regression, adjusting for covariates as described above.

The intervention effect will be assessed by analysis of subgroups defined by severity of COPD, smoking status, hospital admission in the previous year, attendance on a pulmonary rehabilitation course in the previous year and the presence or absence of live-in support. The full detailed statistical analysis plan, including any prespecified subgroup and sensitivity analyses, will be prepared before the final analysis by the trial statistician.

### Embedded qualitative process evaluation

An embedded qualitative study will involve individual interviews with a subgroup of up to 30 patients in the intervention group invited to take part. Interviews will take place at baseline (after patients have been randomised and prior to the delivery of the EDGE platform-based intervention) and after the 12-month visit, and will be carried out by a qualitative researcher. The aim of the baseline interview is to explore patients’ current self-management strategies, while the second interview will focus on how the EDGE platform intervention has impacted on their self-management of COPD and explore issues of acceptability, everyday use and usability.

A maximum variation sample in terms of age, gender, employment status, care support, severity of COPD, exacerbation frequency and experience of using computer or smart phone technology will be sought. Interviews will be audio-recorded and transcribed verbatim. NVivo will be used to facilitate organisation and analysis of data. Analytical procedures will follow grounded theory methods,^27^ including double coding by a second qualitative researcher to ensure rigour. The EDGE COPD trial will be carried out in conformance with the principles of the current version of the Declaration of Helsinki and the other regulations in force.

## Discussion

This trial is designed to assess the extent to which a telehealth platform designed to support self-management improves quality of life for people with COPD. Reduced quality of life is an important aspect of COPD that has been shown to be responsive to group and one-to-one interventions.^7^ This trial is powered to show a clinically important effect and provide data from which to design further trials to explore cost-effectiveness and potential reduction in hospital admissions.

The trial intervention uses a novel implementation of telehealth using a non-proprietary tablet computer designed to be integrated into day-to-day life and clinical care. In addition to being non-obtrusive, it provides, at relatively low cost, in relation to previous telehealth systems, facilities for monitoring, communication, self-management support and education delivery. The development of the system was carried out iteratively using best practice to involve patients, engineers and clinicians in repeated testing and assessment.^28^

The underlying approach in implementing telehealth within this trial is to provide a system focused around the needs of the patient with collection of data that can be analysed over a period of time and used to inform future management. Although future implementations of this system may allow real-time alerts to inform management, consistent experience to date with existing commercial telehealth systems has been that the high rates of false-positive alerts have been unacceptable to clinicians and patients. This trial will provide data to evaluate the potential of the patient-specific tailored alerts available in the system for implementation in practice.

The current evaluation has a number of strengths in comparison with previous trials of telehealth systems. In particular, the information provided by the platform is timely, and immediately available, but does not provide an overwhelming flow of data. The platform has been evaluated for acceptability and feasibility in a cohort study over a period of 6 months in which the tablets were used daily by over 80% of participants. The process of completing diaries and measuring oxygen saturation takes less than 3 min, with an average of five video segments viewed by each participant during their 6-month participation in the cohort study. A qualitative process evaluation adds to the strength of the design. The unbalanced allocation will provide additional power to examine the effect of the EDGE platform across a wide range of participants. The trial, however, is not sufficiently large to provide a detailed cost-effectiveness evaluation, or to provide sufficient power to demonstrate clinically important differences between intervention and usual care groups for hospital admission rates. The trial is due to report in September 2015.

## Supplementary Material

Author's manuscript
