# Supplementary material for: Self-management support using an Internet-linked tablet computer (the EDGE platform)-based intervention in chronic obstructive pulmonary disease: protocol for the EDGE-COPD randomised controlled trial
Source: BMJ Open. 2014 Jan 8;4(1):e004437. doi: 10.1136/bmjopen-2013-004437 (PMC3902407; doi:10.1136/bmjopen-2013-004437)
Supplement: Author's manuscript [file bmjopen-2013-004437.draft_revisions.pdf]

# Self-management support using an internet-linked tablet computer (the EDGE platform) based intervention in Chronic Obstructive Pulmonary Disease: protocol for the EDGE-COPD randomised controlled trial


| | |
|---|---|
| Journal: | *BMJ Open* |
| Manuscript ID: | bmjopen-2013-004437 |
| Article Type: | Protocol |
| Date Submitted by the Author: | 08-Nov-2013 |
| Complete List of Authors: | Farmer, Andrew; University of Oxford, Department of Primary Care Health Sciences<br>Toms, Christy; University of Oxford, Department of Primary Care Health Sciences<br>Hardinge, Maxine; Oxford University Hospitals Trust, Department of Respiratory Medicine<br>Williams, Veronika; University of Oxford, Department of Primary Care Health Sciences<br>Rutter, Heather; Oxford Health NHS Foundation Trust,<br>Tarassenko, Lionel; University of Oxford, Institute of Biomedical Engineering |
| <b>Primary Subject Heading</b>: | General practice / Family practice |
| Secondary Subject Heading: | Respiratory medicine |
| Keywords: | Telemedicine < BIOTECHNOLOGY & BIOINFORMATICS, PRIMARY CARE, RESPIRATORY MEDICINE (see Thoracic Medicine) |
| | |


SCHOLARONE™ Manuscripts

For peer review only

# Self-management support using an internet-linked tablet computer (the EDGE platform) based intervention in Chronic Obstructive Pulmonary Disease: protocol for the EDGE-COPD randomized controlled trial.

Andrew Farmer[1*], Christy Toms[1], Maxine Hardinge[2], Veronika Williams[1], Heather Rutter[3], Lionel Tarassenko[4].

* Corresponding author
23-38 Hythe Bridge Street, Oxford OX1 2ET
andrew.farmer@phc.ox.ac.uk tel: 01865 289300 fax: 01865 289412

1. Department of Primary Care Health Sciences, University of Oxford, Oxford, UK
2. Oxford University Hospitals NHS Trust, Oxford, UK
3. Oxford Health NHS Foundation Trust, Oxford, UK
4. Institute of Biomedical Engineering, University of Oxford, Oxford, UK

**Keywords:** COPD, Telemedicine, primary care, respiratory medicine.

**Word Count:** 3642

For peer review only

## ABSTRACT

**Introduction:** The potential for telehealth-based interventions to provide remote support, education and improve self-management for long term conditions is increasingly recognised. This trial aims to determine whether an intervention delivered through an easy-to-use tablet computer can improve the quality of life of patients with chronic obstructive pulmonary disease (COPD) by providing personalised self-management information and education.

**Methods and analysis:** The sElf management anD support proGrammE (EDGE) for COPD is a multi-centre, randomised controlled trial designed to assess the efficacy of an internet-linked tablet computer based intervention (the EDGE platform) in improving quality of life in patients with moderate to very severe COPD compared with usual care. Eligible patients are randomly allocated to receive the tablet computer based intervention or usual care in a 2:1 ratio using a web-based randomisation system. Participants are recruited from respiratory outpatient clinics and pulmonary rehabilitation courses as well as from those recently discharged from hospital with a COPD-related admission and from primary care clinics. Participants allocated to the tablet computer based intervention complete a daily symptom diary and record clinical symptoms using a Bluetooth-linked pulse oximeter. Participants allocated to receive usual care are provided with all the information given to those allocated to the intervention but without the use of the tablet computer or the facility to monitor their symptoms or physiological variables. The primary outcome of quality of life is measured using the St George's respiratory questionnaire for COPD patients (SGRQ-C) baseline, 6 and 12 months. Secondary outcome measures are recorded at these intervals in addition to 3 months.

**Ethics and dissemination:** The Research Ethics Committee for Berkshire – South Central has provided ethical approval for the conduct of the study in the recruiting regions. The results of the study will be disseminated through peer review publications and conference presentations.

**Trial registration:** Current controlled trials ISRCTN40367841

For peer review only

# Article Summary

### Article focus

- This protocol paper describes a randomised controlled trial of a novel implementation of telehealth in improving quality of life in patients with Chronic Obstructive Pulmonary Disease.

### Key messages

- The study aims to explore the effectiveness of a mobile, tablet computer based intervention incorporating an individualised self management package in improving patients' quality of life.
- The intervention has been specifically designed to be easily incorporated into day-to-day life and clinical care management.

### Strengths and limitation of this study

- The study is powered to examine quality of life outcomes and uses an unbalanced allocation to examine the effect of the EDGE platform across a wide range of participants.
- The study is not sufficiently large enough to provide a detailed cost-effectiveness evaluation, or to provide sufficient power to demonstrate clinically important differences between intervention and usual care groups for hospital admission rates.

For peer review only

# BACKGROUND

Chronic obstructive pulmonary disease (COPD) is an important cause of morbidity and mortality worldwide responsible for three million deaths globally per year. [1] In the UK the total annual estimated cost of COPD to the NHS is over £800 million, with over half of this attributable to hospital-based care,[2] and the impact of COPD to the health-related quality of life of patients is well established. [3, 4] There is now promising evidence that training and support for patients in the self management of their condition improves quality of life and can reduce unplanned hospital admissions. [5,6] However, the results of individual studies are mixed, [7] and the challenge remains to identify those for whom different forms of self-management are suitable and to develop and optimise ways of delivering available interventions to maximise effectiveness and safety.

Use of the converging computer and communication technologies in the form of telehealth-based interventions offers a means of helping patients monitor their condition, providing support in interpreting data, providing a means of delivering individually tailored education and treatment plans, and allowing clinicians to monitor long-term trends and identify short term safety issues.

Evaluation of telehealth-based interventions can be complex with a requirement for considering multiple perspectives within the evaluation [8] For a successful evaluation there is a need to improve the delivery of clinical care, control the workload placed on healthcare professionals, and to provide cost effective improvements in treatment outcomes.

Systematic reviews of telehealth in COPD provide evidence to support continuing research, [9] but recent large-scale evaluations of telehealth-based treatment programmes have not shown convincing evidence of effectiveness. [10,11] The challenge remains to ensure that the telehealth systems can be easily used by patients and that the systems integrated into the health care system are acceptable to clinicians and patients . Criticism of previous systems has included difficulty with data entry, leading to lifestyle restriction, unreliability of monitoring devices, and lack of integration with an individual patient's day-to day life.

Our group has long experience of developing and testing health care interventions based on the use of mobile phones and their integration into clinical care delivery. Mobile phones or tablet computers with a Subscriber Identity Module (SIM) card provide a platform in which communication and computing technologies are sustainably integrated, and provide a platform for communicating with monitoring devices. We have recently carried out a cohort

study in which we have shown that a tablet-computer based system for supporting patients with COPD is acceptable and feasible.[12]

We have therefore set out to determine the efficacy of an internet-linked tablet computer based intervention (the EDGE - sElf management anD support proGrammE - platform), with patients with moderate to very severe COPD, in improving quality of life measured with the St Georges Respiratory questionnaire for COPD patients (SGRQ-C) in comparison with standardised usual care. In addition we will collect data on morbidity, mortality and hospital admissions to inform the design of future evaluations of the system.

# METHODS

## Trial Design

The sElf management anD support proGrammE (EDGE) for COPD is a multi-centre, randomised controlled trial of twelve-month duration. Patients are individually randomised to receive either an internet-linked tablet computer (the EDGE platform) based intervention or standardised usual care in a 2:1 allocation ratio (Figure 1).

## Participants

*Eligibility criteria for participants*

Eligible patients are those aged ≥40 years of age with a confirmed diagnosis of chronic obstructive pulmonary disease (COPD) defined as a forced one second expiratory volume (FEV1) post-bronchodilation of <80% and a predicted ratio of FEV1 to forced vital capacity (FVC) of <0.70. A clinical decision of trial suitability for patients who are unable, for clinical reasons, to provide a spirometry reading at full assessment is sufficient for eligibility if the patient has prior clinical evidence of COPD (e.g. obstructive spirometry within the last 10 years or radiological evidence of emphysema). Patients are required to be registered with a general practitioner and have had an exacerbation of COPD requiring home treatment or hospital admission in the previous year or have been referred for pulmonary rehabilitation.

Patients are not eligible for trial participation if they have a smoking history of <10 pack years, an MRC dyspnoea score <2, other significant lung disease or chronic heart failure (defined by the New York Heart Association classification system as severe (grade IV)) or a

For peer review only

life expectancy of <3 months. Eligible patients must be able to provide informed consent, to complete the trial questionnaires and not be cognitively impaired. Patients living in areas without access to a mobile phone network able to provide access to the Internet, and thus unable to transmit and receive data are not eligible to enter the trial.

*Setting*

Participants are recruited from patients attending respiratory hospital outpatient clinics and pulmonary rehabilitation courses in the adjacent counties of Oxfordshire and Berkshire, United Kingdom (UK). In addition, eligible patients are identified from primary care clinics and from those recently (within the preceding two weeks) discharged from hospital following a COPD-related admission.

## Trial interventions

*Intervention development and specification*

We developed and tested an intervention to support patients with COPD in monitoring their health and to provide information and education about their condition based on use of an internet-linked tablet computer (the EDGE platform). Based on open architecture application software, the EDGE platform was developed to allow integration within clinical care by a team of clinicians and engineers working with patients. The platform was refined in a six-month cohort study with a group of COPD patients selected using eligibility criteria matching those of the trial. Key principles underlying the development of the platform include ease of use for a group of participants less experienced with computers (large icons, no keyboard needed for data entry) and ensuring data quality (tablet based instructions to ensure ease of use, development of algorithms to assure data quality).

The EDGE platform intervention incorporates a daily symptom diary consisting of a series of standard questions about symptoms based on previous trial protocols.[2,13,14] Questions include general well-being, cough, breathlessness, sputum (quantity produced and colour) and use of medications. A thirty-second period of data transmission using a Bluetooth-enabled pulse oximeter with finger probe allows daily collection of pulse rate and oxygen saturation data. Every four weeks, beginning two weeks after initial use, the platform presents the four-item Patient Health Questionnaire (PHQ-4) screening measure[5,15] and if either the depression or anxiety components score is ≥3 then the relevant full questionnaire (depression (PHQ-8,[6,16]) or anxiety (GAD7,[7,17]) is presented for completion.

The EDGE platform also includes software modules to provide patients with additional support for self-management of their condition. These include (i) personalised plans for self-management and treating an exacerbation of their condition (ii) brief-video clips and text-based material providing additional information about COPD and treatments (including medicines use and inhaler technique), and educational advice on managing COPD, smoking cessation, diet, physical activity, and mood management (depression and anxiety), and (iii) the facility to receive a brief message from their respiratory nurse. Prompts for accessing the physical activity and mood management information are included for patients reporting higher scores on the depression and anxiety screening measures.

The data entered onto the EDGE platform is transmitted immediately to a server hosted behind NHS firewalls. The data held on the server is reviewed at no less than four-day intervals by a clinician to identify technical problems and review clinical data received.

In an initial six-week period of use, EDGE-platform users complete the symptom diary and record their oxygen saturation and heart rate with the pulse oximeter on a daily basis. Following this initial run-in period of use, the distributions of values for the oxygen saturation, heart rate and symptom scores are calculated for the run-in period for each participant. The 95$^{th}$ centile is computed for each distribution and used as the threshold for the participant's safety alert for that parameter. Every time the participant-specific threshold for a parameter is crossed after the run-in period, this safety alert is displayed on the web-based record for that participant.

*The EDGE platform based intervention*

Participants allocated to receive the Internet-linked tablet computer (EDGE platform)-based intervention are provided with an Android tablet computer (Samsung Galaxy Tab) running the application software and Bluetooth-enabled oximeter probe (Nonin, PureSAT, 956OBT, Nonin Medical Inc, Plymouth Minnesota). Participants are briefly instructed on the use of the EDGE platform by the research nurse and are provided with a brief information booklet giving details of its use. Participants continue to input their symptom data and clinical recordings daily throughout the duration of the trial. Patients are informed that the EDGE system is not a replacement for their usual clinical care, and that in the event of deterioration in their health they should contact their general practitioner or community respiratory nurse as usual. However, data are reviewed by a clinician at no less than four-day intervals, and changes in heart rate, oxygen saturation or symptom score that cross the participant's

For peer review only

specific threshold for that parameter and persist for more than four days are followed up with a phone call to either the patient, general practitioner or community respiratory nurse as appropriate. If depression or anxiety scores equal or exceed a threshold of 10, then the general practitioner is informed by letter.

*The standardised usual care intervention*

Participants allocated to receive standardised usual care are provided with all the information given to those allocated to use the EDGE platform, but without the use of a tablet computer or the facility for daily monitoring of symptoms and physiological variables. The research nurse provides participants with leaflets based on those currently produced by the Oxfordshire Community Respiratory service. Personalised information intended to help patients understand their condition includes information about how to use their medications and when they should be used, a self-management plan with written guidelines on what to do and who to contact if they experience an exacerbation and dietary advice is provided. If the participant has not attended a pulmonary rehabilitation course they are invited to do so.

**Outcomes**

Trial outcome measures will be recorded as indicated in Table 1.

*Primary outcome measure*

The primary outcome is the St George's Respiratory Questionnaire for COPD patients (SGRQ-C). [8,18] used to assess quality of life in patients with moderate to severe COPD.

*Secondary outcome measures*

The following secondary endpoints will be used to evaluate the impact of the intervention in comparison to usual care (i) impact on hospital admissions (number of admissions and days out of hospital) and deaths; (ii), the number of recorded exacerbations defined as episodes in which antibiotics or oral steroids were prescribed or in which the patients were seen in the Accident and Emergency Department and/or admitted to hospital as a result of a respiratory episode; (iii) time to first exacerbation; (iv) beliefs about respiratory medicine use measured with the Beliefs about Medicines Questionnaire; [9,19] (v) self-reported medication use measured with the Medication Adherence Report Schedule; [10,11,19] (vi) self-reported smoking cessation; (vii) mood measured with the standard checklist 20-item questionnaire (SCL-20)

For peer review only - http://bmjopen.bmj.com/site/about/guidelines.xhtml

for depression[20] and the standard checklist 10-item anxiety measure (SCL-10A); [21] and (viii) health status measured with the EuroQol 5-Dimension Questionnaire (EQ-5D). [22]

Details of number and duration of hospital admissions will be measured by self-report and confirmed where possible by a review of hospital discharge letters and central hospital admissions data. Records of deaths will be obtained from general practices and further details obtained where necessary from hospital records.

Details of exacerbations of COPD will be recorded on a record form held by all participants. This will include number, severity, medications prescribed and outcomes to enable, where possible, details to be confirmed by review of medical records or, if deterioration results in admission, review of hospital records.

Costs of healthcare will be identified using a brief questionnaire to obtain self-reported information about visits to general practitioners. Standard costs will be used for hospital admissions and prescribed respiratory medicine.

**Sample size**

The sample size calculations are based on the numbers of patients required to demonstrate a mean difference of 6.6 on the St George's Respiratory Questionnaire between the two trial groups to which participants are allocated, over a twelve-month period (equivalent to 7.3 on SGRC-C).[18] Although there is limited data on this outcome in settings using computer-based interventions, we have estimated the standard deviation at 12.7 based on a study using the SGRC. [23] A trial using these estimates with a power of 90% and significance level of 0.05 (2-sided), with 2:1 allocation between intervention and usual care and allowing for 10% loss to follow-up would require 165 patients.

We also have 98% power to identify a difference in admissions to hospital at 3-months based on effect sizes of previous intensive interventions with this group of patients,[24] and 52% power to detect a difference in admissions at twelve months based on a systematic review of interventions in COPD.[25] In both cases a 5% loss to follow-up has been assumed.

**Randomisation**

Participants are randomised with an allocation ratio of 2:1 intervention to usual care using Sortition V1.2 (an online, web-based randomisation system developed by the Primary Care Clinical Trials Unit at the University of Oxford). A computer schedule based on recruiting site (Oxfordshire or Berkshire), age (≤70 years or >70 years), gender, COPD severity

For peer review only

(moderate or severe/very severe) and current smoking status (yes or no) is used to minimise imbalance between the groups,[26] and is monitored by an independent statistician. The research nurse randomises the patient by accessing Sortition using a web-browser on a tablet computer at the assessment visit only after completion of consent procedures and baseline measurements, including completion of the SGRC-C.

Self-completed outcome measures at six and twelve months are completed without guidance by the research team and prior to any further assessment or discussion of clinical care. Research and clinical teams are trained in the potential for measures to be biased by their interactions with participants. A record of all contacts with trial participants is kept to examine potential for interactions with patients not specified in the trial protocol.

## Trial Procedures

### *Recruitment*

Potentially eligible patients are identified from those discharged from hospital following a COPD-related admission, from respiratory hospital outpatient clinics, pulmonary rehabilitation courses and from primary care clinics, and are sent an invitation to participate in the trial. The invitation includes a patient information booklet, a reply slip and pre-paid envelope. Patients interested in participating are asked to return their reply slips by post to the research team. The research nurse then contacts the patient by telephone to arrange an initial assessment visit. At this visit eligibility is confirmed, written informed consent obtained and baseline data collected for those consenting to participate.

All participants are assessed at baseline by a clinician and complete self-completed measures prior to randomisation and intervention allocation. The use of medication by participants is recorded at the baseline and follow-up assessment visits. Information collected includes type, dose and frequency of COPD medication (tablets, inhalers) as well as a list of other medication taken. A detailed smoking history is taken at the baseline assessment visit; self-reported smoking status is recorded at subsequent assessments.

Patients remain in the trial for 12 months with assessments at a baseline visit, three, six and twelve months. The primary outcome measure is collected at baseline, and six and twelve months after randomisation. Secondary outcome measures are collected at baseline, three, six and twelve months. The three-month visit is a telephone contact, the six and twelve-month

For peer review only

visits are carried out either at home or at clinic whichever is more convenient for the participant.

**Statistical methods**

The principal comparisons will be performed on an intention-to-treat basis. The trial results will be presented as comparative summary statistics (difference in response rates or means) with 95% confidence intervals. If appropriate, and depending on the distribution of the continuous outcome measures, a linear mixed-effects model will be used to analyse SGRQ-C over the twelve month period of the trial, adjusting for baseline value and minimisation covariates. Treatment-time interaction will be included in the model to assess the treatment effect at twelve months. We will formally assess the distribution of the change from baseline for evidence of departure from normality. If necessary, data will either be transformed or analysed using a non-parametric equivalent. The nature and mechanism for the missing outcomes will be investigated, though mixed effects models implicitly account for data missing at random. Sensitivity analyses will be carried out to examine the robustness of the results with different assumptions about departures from randomisation policies, and handling of missing data. Binary outcomes will be analysed using log-binomial regression, adjusting for covariates as described above.

A full detailed statistical analysis plan, including any pre-specified subgroup and sensitivity analyses, will be prepared before the final analysis by the trial statistician.

**Embedded qualitative process evaluation**

An embedded qualitative study will involve individual interviews with a sub group of up to 30 patients in the intervention group invited to take part. Interviews will take place at baseline (after patients have been randomised and prior to the delivery of the EDGE platform based intervention) and after the 12-month visit, and will be carried out by a qualitative researcher. The aim of the baseline interview is to explore patients' current self-management strategies, whilst the second interview will focus on how the EDGE platform intervention has impacted on their self-management of COPD, and explore issues of acceptability, everyday use and usability.

A maximum variation sample in terms of age, gender, employment status, care support, severity of COPD, exacerbation frequency and experience of using computer or smart phone technology will be sought. Interviews will be audio-recorded and transcribed verbatim.

For peer review only

NVivo© will be used to facilitate organisation and analysis of data. Analytical procedures will follow grounded theory methods, [27] including double coding by a second qualitative researcher to ensure rigour.

**Ethical approvals**

The EDGE COPD trial will be carried out in conformance with the principles of the current version of the Declaration of Helsinki and the other regulations in force. Ethical approval has been received from the South Central - Berkshire Research Ethics Committee of the United Kingdom National Research Ethics Service (Ethics Ref: 12/SC/0437). The trial analysis and reporting will be independent of the trial funder and sponsor. The trial is registered with Current Controlled Trials ISRCTN40367841.

## DISCUSSION

This trial is designed to assess the extent to which a telehealth platform designed to support self-management improves quality of life for people with chronic obstructive pulmonary disease. Reduced quality of life is an important aspect of COPD that has been shown to be responsive to group and one-to-one interventions. [7] This trial is powered to show a clinically important effect, and provide data from which to design further trials to explore cost-effectiveness and potentially reduction in hospital admissions.

The trial intervention uses a novel implementation of telehealth using a non-proprietary tablet computer designed to be integrated into day-to-day life and clinical care. In addition to being non-obtrusive it provides, at relatively low cost in relation to previous telehealth systems, facilities for monitoring, communication, self-management support and education delivery. The development of the system was carried out iteratively using best practice to involve patients, engineers and clinicians in repeated testing and assessment. [28]

The underlying approach to implementing telehealth within this trial is to provide a system focussed around the needs of the patient with collection of data that can be analysed over a period of time and used to inform future management. Although future implementations of this system may allow real-time alerts to inform management, consistent experience to date with existing commercial telehealth systems has been that the high rates of false-positive alerts have been unacceptable to clinicians and patients. This trial will provide data to

For peer review only

evaluate the potential of the patient-specific tailored alerts available in the system for implementation in practice.

The current evaluation has a number of strengths in comparison with previous trials of telehealth systems. In particular the information provided by the platform is timely, and immediately available, but does not provide an overwhelming flow of data. The platform has been evaluated for acceptability and feasibility in a cohort study over a period of six months in which the tablets were used daily by over 80% of participants. The process of completing diaries and measuring oxygen saturation takes less than three minutes, with an average of five video segments viewed by each participant during their six-month participation in the cohort study. A qualitative process evaluation adds to the strength of the design. The unbalanced allocation will provide additional power to examine the effect of the EDGE platform across a wide range of participants. The trial however, is not sufficiently large to provide a detailed cost-effectiveness evaluation, nor to provide sufficient power to demonstrate clinically important differences between intervention and usual care groups for hospital admission rates. The trial is due to report in September 2015.

## ACKNOWLEDGEMENTS

The authors would like to acknowledge the EDGE COPD Trial group consisting of, in addition to the authors of this manuscript, Dr Jonathan Price, Dr Carl Heneghan and Dr Gari Clifford who provided clinical and scientific input into the design of the intervention and trial and the trial engineering team including Dr Oliver Gibson, Dr Carmelo Velardo and Dr Syed Ahmar Shah who developed and delivered the EDGE platform. Grace Robinson (respiratory consultant and principal investigator), Katherine Thomas (respiratory clinician) and Jacqui Webb (research nurse) lead recruitment at the Royal Berkshire Hospital Department of Respiratory Medicine.

For peer review only

## FOOTNOTES

**Contributors:** LT and AF conceived the original idea for the trial and all authors were involved in the development of the intervention and design of the trial. AF, assisted by CT drafted the initial manuscript. All authors commented on subsequent drafts and approved the final version.

**Funding:**

This work was supported by the Wellcome Trust and Department of Health, Health Innovation Challenge Fund; grant number HICF-1010-032.

LT and AF receive funding from the Oxford NIHR BRC. AF is an NIHR Senior Investigator.

**Competing interests:**

None

For peer review only

# REFERENCES


1 World Health Organization. *The global burden of disease: 2004 update*. Geneva: : WHO 2008.

2 National Clinical Guideline Centre. *Chronic obstructive pulmonary disease: Management of chronic obstructive pulmonary disease in adults in primary and secondary care*. London: : NICE 2010.

3 Okubadejo AA, O'Shea L, Jones PW, *et al.* Home assessment of activities of daily living in patients with severe chronic obstructive pulmonary disease on long-term oxygen therapy. *European Respiratory Journal* 1997;**10**:1572–5.

4 Ståhl E, Lindberg A, Jansson S-A, *et al.* Health-related quality of life is related to COPD disease severity. *HealthQualLifeOutcomes* 2005;**3**:56–6.

5 Bourbeau J, Julien M, Maltais F, *et al.* Reduction of hospital utilization in patients with chronic obstructive pulmonary disease: a disease-specific self-management intervention. *ArchInternMed* 2003;**163**:585–91.

6 Rea HH, McAuley SS, Stewart AA, *et al.* A chronic disease management programme can reduce days in hospital for patients with chronic obstructive pulmonary disease. *Intern Med J* 2004;**34**:608–14.

7 Effing T, Monninkhof EM, van der Valk PDLPM, *et al.* Self-management education for patients with chronic obstructive pulmonary disease. *Cochrane Database SystRev* 2007;:CD002990.

8 Greenhalgh T, Procter R, Wherton J, *et al.* The organising vision for telehealth and telecare: discourse analysis. *BMJ Open* 2012;**2**:e001574–4.

9 Polisena J, Tran K, Cimon K, *et al.* Home telehealth for chronic obstructive pulmonary disease: a systematic review and meta-analysis. *JTelemedTelecare* 2010;**16**:120–7.

10 Steventon A, Bardsley M, Billings J, *et al.* Effect of telehealth on use of secondary care and mortality: findings from the Whole System Demonstrator cluster randomised trial. *BMJ* 2012;**344**:e3874–4.

11 Fairbrother PP, Pinnock HH, Hanley JJ, *et al.* Continuity, but at what cost? The impact of telemonitoring COPD on continuities of care: a qualitative study. *Prim Care Respir J* 2012;**21**:322–8.

12 M-Health for long term management of COPD. The Kings Fund, London: 2013.

13 Time course and recovery of exacerbations in patients with chronic obstructive pulmonary disease. 2000;**161**:1608–13.

14 Bucknall CE, Miller G, Lloyd SM, *et al.* Glasgow supported self-management trial (GSuST) for patients with moderate to severe COPD: randomised controlled trial. *BMJ* 2012;**344**:e1060–0.

15 A 4-item measure of depression and anxiety: Validation and standardization of the Patient Health Questionnaire-4 (PHQ-4) in the general population. 2010;**122**:86–95.

16 Kroenke K, Strine TW, Spitzer RL, *et al.* The PHQ-8 as a measure of current depression in the general population. *Journal of Affective Disorders* 2009;**114**:163–73.

17 A brief measure for assessing generalized anxiety disorder: the GAD-7. 2006;**166**:1092–7.


For peer review only

18 Meguro M, Barley EA, Spencer S, *et al.* Development and Validation of an Improved, COPD-Specific Version of the St. George Respiratory Questionnaire. *Chest* 2007;**132**:456–63.

19 Horne R, Weinman J, Hankins M. The beliefs about medicines questionnaire: The development and evaluation of a new method for assessing the cognitive representation of medication. *PsycholHealth* 1999;**14**:1–24.

20 Derogatis LRL, Lipman RSR, Covi LL. SCL-90: an outpatient psychiatric rating scale--preliminary report. *Psychopharmacol Bull* 1973;**9**:13–28.

21 The Hopkins Symptom Checklist (HSCL): a self-report symptom inventory. 1974;**19**:1–15.

22 Group E. EuroQol: a new facility for the measurement of health related quality of life. *Health Pol* 2001;**16**:199–208.

23 Welte T, Miravitlles M, Hernandez P, *et al.* Efficacy and Tolerability of Budesonide/Formoterol Added to Tiotropium in Patients with Chronic Obstructive Pulmonary Disease. *Am J Respir Crit Care Med* 2009;**180**:741–50.

24 Seymour JM, Moore L, Jolley CJ, *et al.* Outpatient pulmonary rehabilitation following acute exacerbations of COPD. *Thorax* 2010;**65**:423–8.

25 McLean S, Nurmatov U, Liu JL, *et al.* Telehealthcare for chronic obstructive pulmonary disease. *Cochrane Database SystRev* 2011;:CD007718.

26 Altman DG, Bland JM. Treatment allocation by minimisation. *BMJ* 2005;**330**:843.

27 Charmaz K, Mitchell RG. Grounded theory in Ethnography. In: Atkinson P, Coffey A, Delamont S, *et al.*, eds. *Handbook of ethnography*. London: : SAGE Publications 2001.

28 Campbell NC, Murray E, Darbyshire J, *et al.* Designing and evaluating complex interventions to improve health care. *BMJ* 2007;**334**:455–9.

For peer review only

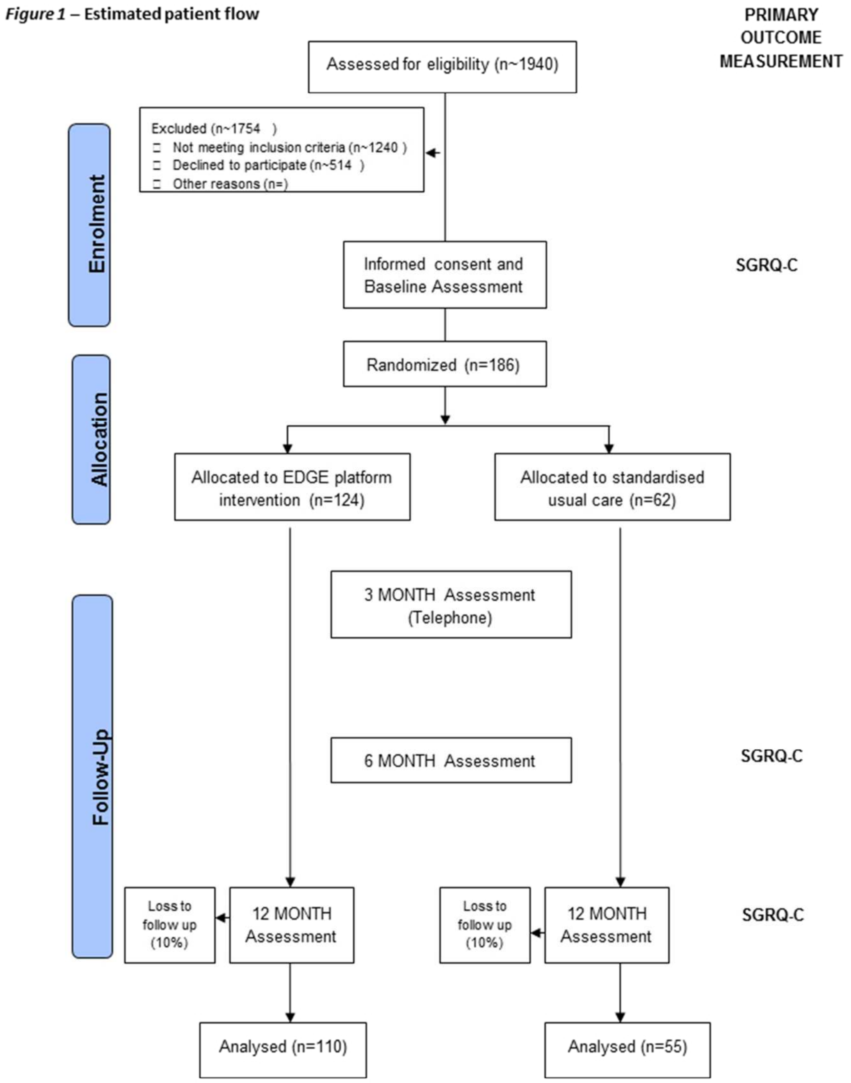


Figure 1 - Estimated Patient Flow
190x254mm (96 x 96 DPI)

| | Baseline assessment visit | Three months | Six months | Twelve months |
|---|---|---|---|---|
| Primary outcome | | | | |
| SGRQ-C (quality of life)[18] | x | | x | x |
| Secondary outcomes | | | | |
| Number of exacerbations | x | x | x | x |
| Time to first exacerbation (days) | | x | x | x |
| Unplanned healthcare contacts | | x | x | x |
| EuroQol 5 Dimension Questionnaire[22] | x | | x | x |
| Self-Completed 20 item depression measure D[20] | x | | x | x |
| Self-Completed 10 item anxiety measure [21] | x | | x | x |
| Beliefs about medicines questionnaire[19] | x | | x | x |
| Medication adherence report schedule[19] | x | | x | x |
| Prescribed medicines | x | x | x | x |
| Smoking status | x | | x | x |
| Health Service usage | x | x | x | x |
| Death | | x | x | x |
| Admission to hospital | x | x | x | x |
| Days out hospital | x | x | x | x |

Table 1: Trial Outcome measures matrix

Table 1 - Trial Outcome measures matrix
190x254mm (96 x 96 DPI)

SPIRIT

STANDARD PROTOCOL ITEMS: RECOMMENDATIONS FOR INTERVENTIONAL TRIALS

SPIRIT 2013 Checklist: Recommended items to address in a clinical trial protocol and related documents*

| Section/item | Item No | Description | Addressed on page number |
|---|---|---|---|
| **Administrative information** | | | |
| Title | 1 | Descriptive title identifying the study design, population, interventions, and, if applicable, trial acronym | 1 |
| Trial registration | 2a | Trial identifier and registry name. If not yet registered, name of intended registry | |
| | 2b | All items from the World Health Organization Trial Registration Data Set | |
| Protocol version | 3 | Date and version identifier | 1 |
| Funding | 4 | Sources and types of financial, material, and other support | 31 |
| Roles and responsibilities | 5a | Names, affiliations, and roles of protocol contributors | 1 |
| | 5b | Name and contact information for the trial sponsor | 1 |
| | 5c | Role of study sponsor and funders, if any, in study design; collection, management, analysis, and interpretation of data; writing of the report; and the decision to submit the report for publication, including whether they will have ultimate authority over any of these activities | |
| | 5d | Composition, roles, and responsibilities of the coordinating centre, steering committee, endpoint adjudication committee, data management team, and other individuals or groups overseeing the trial, if applicable (see Item 21a for data monitoring committee) | 30 |

For peer review only

**Introduction**

| | | | |
|---|---|---|---|
| Background and rationale | 6a | Description of research question and justification for undertaking the trial, including summary of relevant studies (published and unpublished) examining benefits and harms for each intervention | ___7__________ |
| | 6b | Explanation for choice of comparators | ______________ |
| Objectives | 7 | Specific objectives or hypotheses | ___9__________ |
| Trial design | 8 | Description of trial design including type of trial (eg, parallel group, crossover, factorial, single group), allocation ratio, and framework (eg, superiority, equivalence, noninferiority, exploratory) | ___9__________ |

**Methods: Participants, interventions, and outcomes**

| | | | |
|---|---|---|---|
| Study setting | 9 | Description of study settings (eg, community clinic, academic hospital) and list of countries where data will be collected. Reference to where list of study sites can be obtained | ___18__________ |
| Eligibility criteria | 10 | Inclusion and exclusion criteria for participants. If applicable, eligibility criteria for study centres and individuals who will perform the interventions (eg, surgeons, psychotherapists) | ___17-18_______ |
| Interventions | 11a | Interventions for each group with sufficient detail to allow replication, including how and when they will be administered | ___20-21______ |
| | 11b | Criteria for discontinuing or modifying allocated interventions for a given trial participant (eg, drug dose change in response to harms, participant request, or improving/worsening disease) | ____25_________ |
| | 11c | Strategies to improve adherence to intervention protocols, and any procedures for monitoring adherence (eg, drug tablet return, laboratory tests) | ______________ |
| | 11d | Relevant concomitant care and interventions that are permitted or prohibited during the trial | ______________ |
| Outcomes | 12 | Primary, secondary, and other outcomes, including the specific measurement variable (eg, systolic blood pressure), analysis metric (eg, change from baseline, final value, time to event), method of aggregation (eg, median, proportion), and time point for each outcome. Explanation of the clinical relevance of chosen efficacy and harm outcomes is strongly recommended | _____10-12____ |
| Participant timeline | 13 | Time schedule of enrolment, interventions (including any run-ins and washouts), assessments, and visits for participants. A schematic diagram is highly recommended (see Figure) | ______35_______ |



For peer review only - http://bmjopen.bmj.com/site/about/guidelines.xhtml

| | | | |
|---|---|---|---|
| Sample size | 14 | Estimated number of participants needed to achieve study objectives and how it was determined, including clinical and statistical assumptions supporting any sample size calculations | _____27________ |
| Recruitment | 15 | Strategies for achieving adequate participant enrolment to reach target sample size | ______________ |

**Methods: Assignment of interventions (for controlled trials)**

| | | | |
|---|---|---|---|
| Allocation: | | | |
| Sequence generation | 16a | Method of generating the allocation sequence (eg, computer-generated random numbers), and list of any factors for stratification. To reduce predictability of a random sequence, details of any planned restriction (eg, blocking) should be provided in a separate document that is unavailable to those who enrol participants or assign interventions | _____26________ |
| Allocation concealment mechanism | 16b | Mechanism of implementing the allocation sequence (eg, central telephone; sequentially numbered, opaque, sealed envelopes), describing any steps to conceal the sequence until interventions are assigned | ______________ |
| Implementation | 16c | Who will generate the allocation sequence, who will enrol participants, and who will assign participants to interventions | _____19________ |
| Blinding (masking) | 17a | Who will be blinded after assignment to interventions (eg, trial participants, care providers, outcome assessors, data analysts), and how | ______________ |
| | 17b | If blinded, circumstances under which unblinding is permissible, and procedure for revealing a participant's allocated intervention during the trial | ______________ |

**Methods: Data collection, management, and analysis**

| | | | |
|---|---|---|---|
| Data collection methods | 18a | Plans for assessment and collection of outcome, baseline, and other trial data, including any related processes to promote data quality (eg, duplicate measurements, training of assessors) and a description of study instruments (eg, questionnaires, laboratory tests) along with their reliability and validity, if known. Reference to where data collection forms can be found, if not in the protocol | _____20-23_____ |
| | 18b | Plans to promote participant retention and complete follow-up, including list of any outcome data to be collected for participants who discontinue or deviate from intervention protocols | ______25_______ |



For peer review only - http://bmjopen.bmj.com/site/about/guidelines.xhtml

| | | | |
|---|---|---|---|
| Data management | 19 | Plans for data entry, coding, security, and storage, including any related processes to promote data quality (eg, double data entry; range checks for data values). Reference to where details of data management procedures can be found, if not in the protocol | _____29-30____ |
| Statistical methods | 20a | Statistical methods for analysing primary and secondary outcomes. Reference to where other details of the statistical analysis plan can be found, if not in the protocol | ______27_______ |
| | 20b | Methods for any additional analyses (eg, subgroup and adjusted analyses) | ______27_______ |
| | 20c | Definition of analysis population relating to protocol non-adherence (eg, as randomised analysis), and any statistical methods to handle missing data (eg, multiple imputation) | ______27_______ |
| **Methods: Monitoring** | | | |
| Data monitoring | 21a | Composition of data monitoring committee (DMC); summary of its role and reporting structure; statement of whether it is independent from the sponsor and competing interests; and reference to where further details about its charter can be found, if not in the protocol. Alternatively, an explanation of why a DMC is not needed | ______30_______ |
| | 21b | Description of any interim analyses and stopping guidelines, including who will have access to these interim results and make the final decision to terminate the trial | _____________ |
| Harms | 22 | Plans for collecting, assessing, reporting, and managing solicited and spontaneously reported adverse events and other unintended effects of trial interventions or trial conduct | ____25-26_____ |
| Auditing | 23 | Frequency and procedures for auditing trial conduct, if any, and whether the process will be independent from investigators and the sponsor | _____________ |
| **Ethics and dissemination** | | | |
| Research ethics approval | 24 | Plans for seeking research ethics committee/institutional review board (REC/IRB) approval | ______1_______ |
| Protocol amendments | 25 | Plans for communicating important protocol modifications (eg, changes to eligibility criteria, outcomes, analyses) to relevant parties (eg, investigators, REC/IRBs, trial participants, trial registries, journals, regulators) | _____________ |



For peer review only - http://bmjopen.bmj.com/site/about/guidelines.xhtml

| | | | |
|---|---|---|---|
| Consent or assent | 26a | Who will obtain informed consent or assent from potential trial participants or authorised surrogates, and how (see Item 32) | _______19_______ |
| | 26b | Additional consent provisions for collection and use of participant data and biological specimens in ancillary studies, if applicable | ______________ |
| Confidentiality | 27 | How personal information about potential and enrolled participants will be collected, shared, and maintained in order to protect confidentiality before, during, and after the trial | ___29-30______ |
| Declaration of interests | 28 | Financial and other competing interests for principal investigators for the overall trial and each study site | ______________ |
| Access to data | 29 | Statement of who will have access to the final trial dataset, and disclosure of contractual agreements that limit such access for investigators | ______________ |
| Ancillary and post-trial care | 30 | Provisions, if any, for ancillary and post-trial care, and for compensation to those who suffer harm from trial participation | ______________ |
| Dissemination policy | 31a | Plans for investigators and sponsor to communicate trial results to participants, healthcare professionals, the public, and other relevant groups (eg, via publication, reporting in results databases, or other data sharing arrangements), including any publication restrictions | ______31_______ |
| | 31b | Authorship eligibility guidelines and any intended use of professional writers | ______________ |
| | 31c | Plans, if any, for granting public access to the full protocol, participant-level dataset, and statistical code | ______________ |
| **Appendices** | | | |
| Informed consent materials | 32 | Model consent form and other related documentation given to participants and authorised surrogates | ______________ |
| Biological specimens | 33 | Plans for collection, laboratory evaluation, and storage of biological specimens for genetic or molecular analysis in the current trial and for future use in ancillary studies, if applicable | ______________ |

*It is strongly recommended that this checklist be read in conjunction with the SPIRIT 2013 Explanation & Elaboration for important clarification on the items. Amendments to the protocol should be tracked and dated. The SPIRIT checklist is copyrighted by the SPIRIT Group under the Creative Commons "Attribution-NonCommercial-NoDerivs 3.0 Unported" license.



For peer review only - http://bmjopen.bmj.com/site/about/guidelines.xhtml

# Self-management support using an internet-linked tablet computer (the EDGE platform) based intervention in Chronic Obstructive Pulmonary Disease: protocol for the EDGE-COPD randomised controlled trial

| | |
|---|---|
| Journal: | *BMJ Open* |
| Manuscript ID: | bmjopen-2013-004437.R1 |
| Article Type: | Protocol |
| Date Submitted by the Author: | 26-Nov-2013 |
| Complete List of Authors: | Farmer, Andrew; University of Oxford, Department of Primary Care Health Sciences<br>Toms, Christy; University of Oxford, Department of Primary Care Health Sciences<br>Hardinge, Maxine; Oxford University Hospitals Trust, Department of Respiratory Medicine<br>Williams, Veronika; University of Oxford, Department of Primary Care Health Sciences<br>Rutter, Heather; Oxford Health NHS Foundation Trust,<br>Tarassenko, Lionel; University of Oxford, Institute of Biomedical Engineering |
| <b>Primary Subject Heading</b>: | General practice / Family practice |
| Secondary Subject Heading: | Respiratory medicine |
| Keywords: | Telemedicine < BIOTECHNOLOGY & BIOINFORMATICS, PRIMARY CARE, RESPIRATORY MEDICINE (see Thoracic Medicine) |
| | |

SCHOLARONE™ Manuscripts

For peer review only - http://bmjopen.bmj.com/site/about/guidelines.xhtml

# Self-management support using an internet-linked tablet computer (the EDGE platform) based intervention in Chronic Obstructive Pulmonary Disease: protocol for the EDGE-COPD randomized controlled trial.

Andrew Farmer[1*], Christy Toms[1], Maxine Hardinge[2], Veronika Williams[1], Heather Rutter[3], Lionel Tarassenko[4].

* Corresponding author
23-38 Hythe Bridge Street, Oxford OX1 2ET
andrew.farmer@phc.ox.ac.uk tel: 01865 289300 fax: 01865 289412

1. Department of Primary Care Health Sciences, University of Oxford, Oxford, UK
2. Oxford University Hospitals NHS Trust, Oxford, UK
3. Oxford Health NHS Foundation Trust, Oxford, UK
4. Institute of Biomedical Engineering, University of Oxford, Oxford, UK

**Keywords:** COPD, Telemedicine, primary care, respiratory medicine.

**Word Count:** 3892



For peer review only - http://bmjopen.bmj.com/site/about/guidelines.xhtml

## ABSTRACT

**Introduction:** The potential for telehealth-based interventions to provide remote support, education and improve self-management for long term conditions is increasingly recognised. This trial aims to determine whether an intervention delivered through an easy-to-use tablet computer can improve the quality of life of patients with chronic obstructive pulmonary disease (COPD) by providing personalised self-management information and education.

**Methods and analysis:** The sElf management anD support proGrammE (EDGE) for COPD is a multi-centre, randomised controlled trial designed to assess the efficacy of an internet-linked tablet computer based intervention (the EDGE platform) in improving quality of life in patients with moderate to very severe COPD compared with usual care. Eligible patients are randomly allocated to receive the tablet computer based intervention or usual care in a 2:1 ratio using a web-based randomisation system. Participants are recruited from respiratory outpatient clinics and pulmonary rehabilitation courses as well as from those recently discharged from hospital with a COPD-related admission and from primary care clinics. Participants allocated to the tablet computer based intervention complete a daily symptom diary and record clinical symptoms using a Bluetooth-linked pulse oximeter. Participants allocated to receive usual care are provided with all the information given to those allocated to the intervention but without the use of the tablet computer or the facility to monitor their symptoms or physiological variables. The primary outcome of quality of life is measured using the St George’s respiratory questionnaire for COPD patients (SGRQ-C) baseline, 6 and 12 months. Secondary outcome measures are recorded at these intervals in addition to 3 months.

**Ethics and dissemination:** The Research Ethics Committee for Berkshire – South Central has provided ethical approval for the conduct of the study in the recruiting regions. The results of the study will be disseminated through peer review publications and conference presentations.

**Trial registration:** Current controlled trials ISRCTN40367841




For peer review only - http://bmjopen.bmj.com/site/about/guidelines.xhtml

## Article Summary

### Article focus

- This protocol paper describes a randomised controlled trial of a novel implementation of telehealth in improving quality of life in patients with Chronic Obstructive Pulmonary Disease.

### Key messages

- The study aims to explore the effectiveness of a mobile, tablet computer based intervention incorporating an individualised self management package in improving patients' quality of life.
- The intervention has been specifically designed to be easily incorporated into day-to-day life and clinical care management.

### Strengths and limitation of this study

- The study is powered to examine quality of life outcomes and uses an unbalanced allocation to examine the effect of the EDGE platform across a wide range of participants.
- The study is not sufficiently large enough to provide a detailed cost-effectiveness evaluation, or to provide sufficient power to demonstrate clinically important differences between intervention and usual care groups for hospital admission rates.

For peer review only

## BACKGROUND

Chronic obstructive pulmonary disease (COPD) is an important cause of morbidity and mortality worldwide responsible for three million deaths globally per year. [1] In the UK the total annual estimated cost of COPD to the NHS is over £800 million, with over half of this attributable to hospital-based care,[2] and the impact of COPD to the health-related quality of life of patients is well established. [3, 4] There is now promising evidence that training and support for patients in the self management of their condition improves quality of life and can reduce unplanned hospital admissions. [5,6] However, the results of individual studies are mixed, [7] and the challenge remains to identify those for whom different forms of self-management are suitable and to develop and optimise ways of delivering available interventions to maximise effectiveness and safety.

Use of the converging computer and communication technologies in the form of telehealth-based interventions offers a means of helping patients monitor their condition, providing support in interpreting data, providing a means of delivering individually tailored education and treatment plans, and allowing clinicians to monitor long-term trends and identify short term safety issues.

Evaluation of telehealth-based interventions can be complex with a requirement for considering multiple perspectives within the evaluation. [8] For a successful evaluation there is a need to improve the delivery of clinical care, control the workload placed on healthcare professionals, and to provide cost effective improvements in treatment outcomes.

Systematic reviews of telehealth in COPD provide evidence to support continuing research, [9] but recent large-scale evaluations of telehealth-based treatment programmes have not shown convincing evidence of effectiveness. [10,11] The challenge remains to ensure that the telehealth systems can be easily used by patients and that the systems integrated into the health care system are acceptable to clinicians and patients . Criticism of previous systems has included difficulty with data entry, leading to lifestyle restriction, unreliability of monitoring devices, and lack of integration within an individual patient's day-to day life.

Our group has long experience of developing and testing health care interventions based on the use of mobile phones and their integration into clinical care delivery. Mobile phones or tablet computers with a Subscriber Identity Module (SIM) card provide a platform in which communication and computing technologies are sustainably integrated, and provide a platform for communicating with monitoring devices. We have recently carried out a cohort



For peer review only - http://bmjopen.bmj.com/site/about/guidelines.xhtml

study in which we have shown that a tablet-computer based system for supporting patients with COPD is acceptable and feasible.[12]

We have therefore set out to determine the efficacy of an internet-linked tablet computer based intervention (the EDGE - sElf management anD support proGrammE - platform), with patients with moderate to very severe COPD, in improving quality of life measured with the St Georges Respiratory questionnaire for COPD patients (SGRQ-C) in comparison with standardised usual care. In addition we will collect data on morbidity, mortality and hospital admissions to inform the design of future evaluations of the system.

## METHODS

### Trial Design

The sElf management anD support proGrammE (EDGE) for COPD is a multi-centre, randomised controlled trial of twelve-month duration. Patients are individually randomised to receive either an internet-linked tablet computer (the EDGE platform) based intervention or standardised usual care in a 2:1 allocation ratio (Figure 1).

### Participants

*Eligibility criteria for participants*

Eligible patients are those aged ≥40 years of age with a confirmed diagnosis of chronic obstructive pulmonary disease (COPD) defined as a forced one second expiratory volume (FEV1) post-bronchodilation of <80% and a predicted ratio of FEV1 to forced vital capacity (FVC) of <0.70. Eligible patients have a smoking pack history >10 pack years and an MRC dyspnoea score ≥ 2.A clinical decision of trial suitability for patients who are unable, for clinical reasons, to provide a spirometry reading at full assessment is sufficient for eligibility if the patient has prior clinical evidence of COPD (e.g. obstructive spirometry within the last 10 years or radiological evidence of emphysema). Patients are required to be registered with a general practitioner and have had an exacerbation of COPD requiring home treatment or hospital admission in the previous year or have been referred for pulmonary rehabilitation.

Patients are not eligible for trial participation if they have other significant lung disease or chronic heart failure (defined by the New York Heart Association classification system as



For peer review only - http://bmjopen.bmj.com/site/about/guidelines.xhtml

severe (grade IV)) or a life expectancy of <3 months. Eligible patients must be able to provide informed consent, to complete the trial questionnaires and not be cognitively impaired. Patients living in areas without access to a mobile phone network able to provide access to the Internet, and thus unable to transmit and receive data are not eligible to enter the trial.

*Setting*

To maximise recruitment of participants to the trial, patients are identified from a variety of settings encompassing primary and secondary care as well as community services. Patients attending respiratory hospital outpatient clinics and pulmonary rehabilitation courses in the adjacent counties of Oxfordshire and Berkshire, United Kingdom (UK) are invited to participate. In addition, eligible patients are identified from primary care clinics and from those recently (within the preceding two weeks) discharged from hospital following a COPD-related admission.

**Trial interventions**

*Intervention development and specification*

We developed and tested an intervention to support patients with COPD in monitoring their health and to provide information and education about their condition based on use of an internet-linked tablet computer (the EDGE platform). Based on open architecture application software, the EDGE platform was developed to allow integration within clinical care by a team of clinicians and engineers working with patients. The platform was refined in a six-month cohort study with a group of COPD patients selected using eligibility criteria matching those of the trial. Key principles underlying the development of the platform include ease of use for a group of participants less experienced with computers (large icons, no keyboard needed for data entry) and ensuring data quality (tablet based instructions to ensure ease of use, development of algorithms to assure data quality).

The EDGE platform intervention incorporates a daily symptom diary consisting of a series of standard questions about symptoms based on previous trial protocols.[2,13,14] Questions include general well-being, cough, breathlessness, sputum (quantity produced and colour) and use of medications. A thirty-second period of data transmission using a Bluetooth-enabled pulse oximeter with finger probe allows daily collection of pulse rate and oxygen saturation data. Every four weeks, beginning two weeks after initial use, the platform presents



For peer review only - http://bmjopen.bmj.com/site/about/guidelines.xhtml

the four-item Patient Health Questionnaire (PHQ-4) screening measure[5,15] and if either the depression or anxiety components score is ≥3 then the relevant full questionnaire (depression (PHQ-8,[6,16]) or anxiety (GAD7,[7,17]) is presented for completion.

The EDGE platform also includes software modules to provide patients with additional support for self-management of their condition. These include (i) personalised plans for self-management and treating an exacerbation of their condition (ii) brief-video clips and text-based material providing additional information about COPD and treatments (including medicines use and inhaler technique), and educational advice on managing COPD, smoking cessation, diet, physical activity, and mood management (depression and anxiety), and (iii) the facility to receive a brief message from their respiratory nurse. Prompts for accessing the physical activity and mood management information are included for patients reporting higher scores on the depression and anxiety screening measures.

The data entered onto the EDGE platform is transmitted immediately to a server hosted behind NHS firewalls. The data held on the server is reviewed at no less than four-day intervals by a clinician to identify technical problems and review clinical data received.

In an initial six-week period of use, EDGE-platform users complete the symptom diary and record their oxygen saturation and heart rate with the pulse oximeter on a daily basis. Following this initial run-in period of use, the distributions of values for the oxygen saturation, heart rate and symptom scores are calculated for the run-in period for each participant. The 95$^{th}$ centile is computed for each distribution and used as the threshold for the participant's safety alert for that parameter. Every time the participant-specific threshold for a parameter is crossed after the run-in period, this safety alert is displayed on the web-based record for that participant.

*The EDGE platform based intervention*

Participants allocated to receive the Internet-linked tablet computer (EDGE platform)-based intervention are provided with an Android tablet computer (Samsung Galaxy Tab) running the application software and Bluetooth-enabled oximeter probe (Nonin, PureSAT, 956OBT, Nonin Medical Inc, Plymouth Minnesota). Participants are briefly instructed on the use of the EDGE platform by the research nurse and are provided with a brief information booklet giving details of its use. Participants continue to input their symptom data and clinical recordings daily throughout the duration of the trial. Patients are informed that the EDGE system is not a replacement for their usual clinical care, and that in the event of deterioration



For peer review only - http://bmjopen.bmj.com/site/about/guidelines.xhtml

in their health they should contact their general practitioner or community respiratory nurse as usual. However, data are reviewed by a clinician at no less than four-day intervals, and changes in heart rate, oxygen saturation or symptom score that cross the participant's specific threshold for that parameter and persist for more than four days are followed up with a phone call to either the patient, general practitioner or community respiratory nurse as appropriate. If depression or anxiety scores equal or exceed a threshold of 10, then the general practitioner is informed by letter.

*The standardised usual care intervention*

Participants allocated to receive standardised usual care are provided with all the information given to those allocated to use the EDGE platform, but without the use of a tablet computer or the facility for daily monitoring of symptoms and physiological variables. The research nurse provides participants with leaflets based on those currently produced by the Oxfordshire Community Respiratory service. Personalised information intended to help patients understand their condition includes information about how to use their medications and when they should be used, a self-management plan with written guidelines on what to do and who to contact if they experience an exacerbation and dietary advice is provided. If the participant has not attended a pulmonary rehabilitation course they are invited to do so.

**Outcomes**

Trial outcome measures will be recorded as indicated in Table 1.

*Primary outcome measure*

The primary outcome is the St George's Respiratory Questionnaire for COPD patients (SGRQ-C). [8,18] used to assess quality of life in patients with moderate to severe COPD.

*Secondary outcome measures*

The following secondary endpoints will be used to evaluate the impact of the intervention in comparison to usual care (i) impact on hospital admissions (number of admissions and days out of hospital) and deaths; (ii), the number of recorded exacerbations defined as episodes in which antibiotics or oral steroids were prescribed or in which the patients were seen in the Accident and Emergency Department and/or admitted to hospital as a result of a respiratory episode; (iii) time to first exacerbation; (iv) beliefs about respiratory medicine use measured with the Beliefs about Medicines Questionnaire; [9,19] (v) self-reported medication use



For peer review only - http://bmjopen.bmj.com/site/about/guidelines.xhtml

measured with the Medication Adherence Report Schedule; [10,11,19] (vi) self-reported smoking cessation; (vii) mood measured with the standard checklist 20-item questionnaire (SCL-20) for depression[20] and the standard checklist 10-item anxiety measure (SCL-10A); [21] and (viii) health status measured with the EuroQol 5-Dimension Questionnaire (EQ-5D). [22]

Details of number and duration of hospital admissions will be measured by self-report and confirmed where possible by a review of hospital discharge letters and central hospital admissions data. Records of deaths will be obtained from general practices and further details obtained where necessary from hospital records.

Details of exacerbations of COPD will be recorded on a record form held by all participants. This will include number, severity, medications prescribed and outcomes to enable, where possible, details to be confirmed by review of medical records or, if deterioration results in admission, review of hospital records.

Costs of healthcare will be identified using a brief questionnaire to obtain self-reported information about visits to general practitioners. Standard costs will be used for hospital admissions and prescribed respiratory medicine.

**Sample size**

The sample size calculations are based on the numbers of patients required to demonstrate a mean difference of 6.6 on the St George's Respiratory Questionnaire between the two trial groups to which participants are allocated, over a twelve-month period (equivalent to 7.3 on SGRC-C).[18] Although there is limited data on this outcome in settings using computer-based interventions, we have estimated the standard deviation at 12.7 based on a study using the SGRC. [23] A trial using these estimates with a power of 90% and significance level of 0.05 (2-sided), with 2:1 allocation between intervention and usual care and allowing for 10% loss to follow-up would require 165 patients.

We also have 98% power to identify a difference in admissions to hospital at 3-months based on effect sizes of previous intensive interventions with this group of patients,[24] and 52% power to detect a difference in admissions at twelve months based on a systematic review of interventions in COPD.[25] In both cases a 5% loss to follow-up has been assumed.

**Randomisation**

Participants are randomised with an allocation ratio of 2:1 intervention to usual care using Sortition V1.2 (an online, web-based randomisation system developed by the Primary Care



For peer review only - http://bmjopen.bmj.com/site/about/guidelines.xhtml

For peer review only

Clinical Trials Unit at the University of Oxford). A computer schedule based on recruiting site (Oxfordshire or Berkshire), age (≤70 years or >70 years), gender, COPD severity (moderate or severe/very severe) and current smoking status (yes or no) is used to minimise imbalance between the groups,[26] and is monitored by an independent statistician. The research nurse randomises the patient by accessing Sortition using a web-browser on a tablet computer at the assessment visit only after completion of consent procedures and baseline measurements, including completion of the SGRC-C.

Self-completed outcome measures at six and twelve months are completed without guidance by the research team and prior to any further assessment or discussion of clinical care. Research and clinical teams are trained in the potential for measures to be biased by their interactions with participants. A record of all contacts with trial participants is kept to examine potential for interactions with patients not specified in the trial protocol.

## Trial Procedures

### *Recruitment*

Potentially eligible patients are identified from those discharged from hospital following a COPD-related admission, from respiratory hospital outpatient clinics, pulmonary rehabilitation courses and from primary care clinics, and are sent an invitation to participate in the trial. The invitation includes a patient information booklet, a reply slip and pre-paid envelope. Patients interested in participating are asked to return their reply slips by post to the research team. The research nurse then contacts the patient by telephone to arrange an initial assessment visit. At this visit eligibility is confirmed, written informed consent obtained and baseline data collected for those consenting to participate.

All participants are assessed at baseline by a healthcare professional and complete self-completed measures prior to randomisation and intervention allocation. The use of medication by participants is recorded at the baseline and follow-up assessment visits. Information collected includes type, dose and frequency of COPD medication (tablets, inhalers) as well as a list of other medication taken. A detailed smoking history is taken at the baseline assessment visit; self-reported smoking status is recorded at subsequent assessments.

### *Patient follow-up and retention*

Patients remain in the trial for 12 months with assessments at a baseline visit, three, six and twelve months. The primary outcome measure is collected at baseline, and six and twelve



For peer review only - http://bmjopen.bmj.com/site/about/guidelines.xhtml

months after randomisation. Secondary outcome measures are collected at baseline, three, six and twelve months.

The three-month assessment is a telephone contact with patients sent reminders prior to the assessment date. For patients allocated to standardised usual care a reminder is posted prior to the assessment date. For patients receiving the EDGE platform intervention, this reminder is sent in the form of a message to the tablet computer. The six and twelve month visits are carried out either at home or at clinic, with patients receiving postal or electronic reminders prior to the assessment visit date. The researcher ensures at each visit that all outcome measures are complete to ensure maximal follow-up data is collected.

All patients have the right to withdraw from the trial at any point, without providing a reason. Those patients who do withdraw from the trial will be asked if they would be willing to provide follow-up information at the 6 and 12 month assessment points. If patients decline no further information will be collected.

**Statistical methods**

The principal comparisons will be performed on an intention-to-treat basis. The trial results will be presented as comparative summary statistics (difference in response rates or means) with 95% confidence intervals. If appropriate, and depending on the distribution of the continuous outcome measures, a linear mixed-effects model will be used to analyse SGRQ-C over the twelve month period of the trial, adjusting for baseline value and minimisation covariates. Treatment-time interaction will be included in the model to assess the treatment effect at twelve months. We will formally assess the distribution of the change from baseline for evidence of departure from normality. If necessary, data will either be transformed or analysed using a non-parametric equivalent. The nature and mechanism for the missing outcomes will be investigated, though mixed effects models implicitly account for data missing at random. Sensitivity analyses will be carried out to examine the robustness of the results with different assumptions about departures from randomisation policies, and handling of missing data. Binary outcomes will be analysed using log-binomial regression, adjusting for covariates as described above.

The intervention effect will be assessed by analysis of sub-groups defined by severity of COPD, smoking status, hospital admission in the previous year, attendance on a pulmonary rehabilitation course in the previous year and the presence or absence of live-in support. The



For peer review only - http://bmjopen.bmj.com/site/about/guidelines.xhtml

For peer review only

full detailed statistical analysis plan, including any pre-specified subgroup and sensitivity analyses, will be prepared before the final analysis by the trial statistician.

**Embedded qualitative process evaluation**

An embedded qualitative study will involve individual interviews with a sub group of up to 30 patients in the intervention group invited to take part. Interviews will take place at baseline (after patients have been randomised and prior to the delivery of the EDGE platform based intervention) and after the 12-month visit, and will be carried out by a qualitative researcher. The aim of the baseline interview is to explore patients' current self-management strategies, whilst the second interview will focus on how the EDGE platform intervention has impacted on their self-management of COPD, and explore issues of acceptability, everyday use and usability.

A maximum variation sample in terms of age, gender, employment status, care support, severity of COPD, exacerbation frequency and experience of using computer or smart phone technology will be sought. Interviews will be audio-recorded and transcribed verbatim. NVivo© will be used to facilitate organisation and analysis of data. Analytical procedures will follow grounded theory methods, [27] including double coding by a second qualitative researcher to ensure rigour.

**Ethical approvals**

The EDGE COPD trial will be carried out in conformance with the principles of the current version of the Declaration of Helsinki and the other regulations in force. Ethical approval has been received from the South Central - Berkshire Research Ethics Committee of the United Kingdom National Research Ethics Service (Ethics Ref: 12/SC/0437). The trial analysis and reporting will be independent of the trial funder and sponsor. The trial is registered with Current Controlled Trials ISRCTN40367841.

## DISCUSSION

This trial is designed to assess the extent to which a telehealth platform designed to support self-management improves quality of life for people with chronic obstructive pulmonary disease. Reduced quality of life is an important aspect of COPD that has been shown to be responsive to group and one-to-one interventions. [7] This trial is powered to show a clinically


For peer review only - http://bmjopen.bmj.com/site/about/guidelines.xhtml

important effect, and provide data from which to design further trials to explore cost-effectiveness and potentially reduction in hospital admissions.

The trial intervention uses a novel implementation of telehealth using a non-proprietary tablet computer designed to be integrated into day-to-day life and clinical care. In addition to being non-obtrusive it provides, at relatively low cost in relation to previous telehealth systems, facilities for monitoring, communication, self-management support and education delivery. The development of the system was carried out iteratively using best practice to involve patients, engineers and clinicians in repeated testing and assessment. [28]

The underlying approach to implementing telehealth within this trial is to provide a system focussed around the needs of the patient with collection of data that can be analysed over a period of time and used to inform future management. Although future implementations of this system may allow real-time alerts to inform management, consistent experience to date with existing commercial telehealth systems has been that the high rates of false-positive alerts have been unacceptable to clinicians and patients. This trial will provide data to evaluate the potential of the patient-specific tailored alerts available in the system for implementation in practice.

The current evaluation has a number of strengths in comparison with previous trials of telehealth systems. In particular the information provided by the platform is timely, and immediately available, but does not provide an overwhelming flow of data. The platform has been evaluated for acceptability and feasibility in a cohort study over a period of six months in which the tablets were used daily by over 80% of participants. The process of completing diaries and measuring oxygen saturation takes less than three minutes, with an average of five video segments viewed by each participant during their six-month participation in the cohort study. A qualitative process evaluation adds to the strength of the design. The unbalanced allocation will provide additional power to examine the effect of the EDGE platform across a wide range of participants. The trial however, is not sufficiently large to provide a detailed cost-effectiveness evaluation, nor to provide sufficient power to demonstrate clinically important differences between intervention and usual care groups for hospital admission rates. The trial is due to report in September 2015.



For peer review only - http://bmjopen.bmj.com/site/about/guidelines.xhtml

## ACKNOWLEDGEMENTS

The authors would like to acknowledge the EDGE COPD Trial group consisting of, in addition to the authors of this manuscript, Dr Jonathan Price, Dr Carl Heneghan and Dr Gari Clifford who provided clinical and scientific input into the design of the intervention and trial and the trial engineering team including Dr Oliver Gibson, Dr Carmelo Velardo and Dr Syed Ahmar Shah who developed and delivered the EDGE platform. Grace Robinson (respiratory consultant and principal investigator), Katherine Thomas (respiratory clinician) and Jacqui Webb (research nurse) lead recruitment at the Royal Berkshire Hospital Department of Respiratory Medicine.

## FOOTNOTES

**Contributors:** LT and AF conceived the original idea for the trial and all authors were involved in the development of the intervention and design of the trial. AF, assisted by CT drafted the initial manuscript. All authors commented on subsequent drafts and approved the final version.

**Funding:**

This publication presents independent research commissioned by the Health Innovation Challenge Fund (HICF-1010-032), a parallel funding partnership between the Wellcome Trust and the Department of Health. The views expressed in this publication are those of the authors and not necessarily those of the Wellcome Trust or the Department of Health. The trial is sponsored by the University of Oxford. Neither the funders nor the sponsor havebeen involved in the design of the study, the collection, management, analysis and interpretation of data; writing of the report; and the decision to publish trial results. Oversight for these activities resides with the EDGE COPD Trial group.

LT and AF receive funding from the Oxford NIHR BRC. AF is an NIHR Senior Investigator.

**Competing interests:**

None

For peer review only

## REFERENCES


1 World Health Organization. *The global burden of disease: 2004 update*. Geneva: : WHO 2008.

2 National Clinical Guideline Centre. *Chronic obstructive pulmonary disease: Management of chronic obstructive pulmonary disease in adults in primary and secondary care*. London: : NICE 2010.

3 Okubadejo AA, O'Shea L, Jones PW, *et al.* Home assessment of activities of daily living in patients with severe chronic obstructive pulmonary disease on long-term oxygen therapy. *European Respiratory Journal* 1997;**10**:1572–5.

4 Ståhl E, Lindberg A, Jansson S-A, *et al.* Health-related quality of life is related to COPD disease severity. *HealthQualLifeOutcomes* 2005;**3**:56–6.

5 Bourbeau J, Julien M, Maltais F, *et al.* Reduction of hospital utilization in patients with chronic obstructive pulmonary disease: a disease-specific self-management intervention. *ArchInternMed* 2003;**163**:585–91.

6 Rea HH, McAuley SS, Stewart AA, *et al.* A chronic disease management programme can reduce days in hospital for patients with chronic obstructive pulmonary disease. *Intern Med J* 2004;**34**:608–14.

7 Effing T, Monninkhof EM, van der Valk PDLPM, *et al.* Self-management education for patients with chronic obstructive pulmonary disease. *Cochrane Database SystRev* 2007;:CD002990.

8 Greenhalgh T, Procter R, Wherton J, *et al.* The organising vision for telehealth and telecare: discourse analysis. *BMJ Open* 2012;**2**:e001574–4.

9 Polisena J, Tran K, Cimon K, *et al.* Home telehealth for chronic obstructive pulmonary disease: a systematic review and meta-analysis. *JTelemedTelecare* 2010;**16**:120–7.

10 Steventon A, Bardsley M, Billings J, *et al.* Effect of telehealth on use of secondary care and mortality: findings from the Whole System Demonstrator cluster randomised trial. *BMJ* 2012;**344**:e3874–4.

11 Fairbrother PP, Pinnock HH, Hanley JJ, *et al.* Continuity, but at what cost? The impact of telemonitoring COPD on continuities of care: a qualitative study. *Prim Care Respir J* 2012;**21**:322–8.

12 M-Health for long term management of COPD. The Kings Fund, London: 2013.

13 Time course and recovery of exacerbations in patients with chronic obstructive pulmonary disease. 2000;**161**:1608–13.

14 Bucknall CE, Miller G, Lloyd SM, *et al.* Glasgow supported self-management trial (GSuST) for patients with moderate to severe COPD: randomised controlled trial. *BMJ* 2012;**344**:e1060–0.

15 A 4-item measure of depression and anxiety: Validation and standardization of the Patient Health Questionnaire-4 (PHQ-4) in the general population. 2010;**122**:86–95.




For peer review only - http://bmjopen.bmj.com/site/about/guidelines.xhtml

16 Kroenke K, Strine TW, Spitzer RL, *et al.* The PHQ-8 as a measure of current depression in the general population. *Journal of Affective Disorders* 2009;**114**:163–73.

17 A brief measure for assessing generalized anxiety disorder: the GAD-7. 2006;**166**:1092–7.

18 Meguro M, Barley EA, Spencer S, *et al.* Development and Validation of an Improved, COPD-Specific Version of the St. George Respiratory Questionnaire. *Chest* 2007;**132**:456–63.

19 Horne R, Weinman J, Hankins M. The beliefs about medicines questionnaire: The development and evaluation of a new method for assessing the cognitive representation of medication. *PsycholHealth* 1999;**14**:1–24.

20 Derogatis LRL, Lipman RSR, Covi LL. SCL-90: an outpatient psychiatric rating scale--preliminary report. *Psychopharmacol Bull* 1973;**9**:13–28.

21 The Hopkins Symptom Checklist (HSCL): a self-report symptom inventory. 1974;**19**:1–15.

22 Group E. EuroQol: a new facility for the measurement of health related quality of life. *Health Pol* 2001;**16**:199–208.

23 Welte T, Miravitlles M, Hernandez P, *et al.* Efficacy and Tolerability of Budesonide/Formoterol Added to Tiotropium in Patients with Chronic Obstructive Pulmonary Disease. *Am J Respir Crit Care Med* 2009;**180**:741–50.

24 Seymour JM, Moore L, Jolley CJ, *et al.* Outpatient pulmonary rehabilitation following acute exacerbations of COPD. *Thorax* 2010;**65**:423–8.

25 McLean S, Nurmatov U, Liu JL, *et al.* Telehealthcare for chronic obstructive pulmonary disease. *Cochrane Database SystRev* 2011;:CD007718.

26 Altman DG, Bland JM. Treatment allocation by minimisation. *BMJ* 2005;**330**:843.

27 Charmaz K, Mitchell RG. Grounded theory in Ethnography. In: Atkinson P, Coffey A, Delamont S, *et al.*, eds. *Handbook of ethnography*. London: : SAGE Publications 2001.

28 Campbell NC, Murray E, Darbyshire J, *et al.* Designing and evaluating complex interventions to improve health care. *BMJ* 2007;**334**:455–9.

For peer review only



For peer review only - http://bmjopen.bmj.com/site/about/guidelines.xhtml

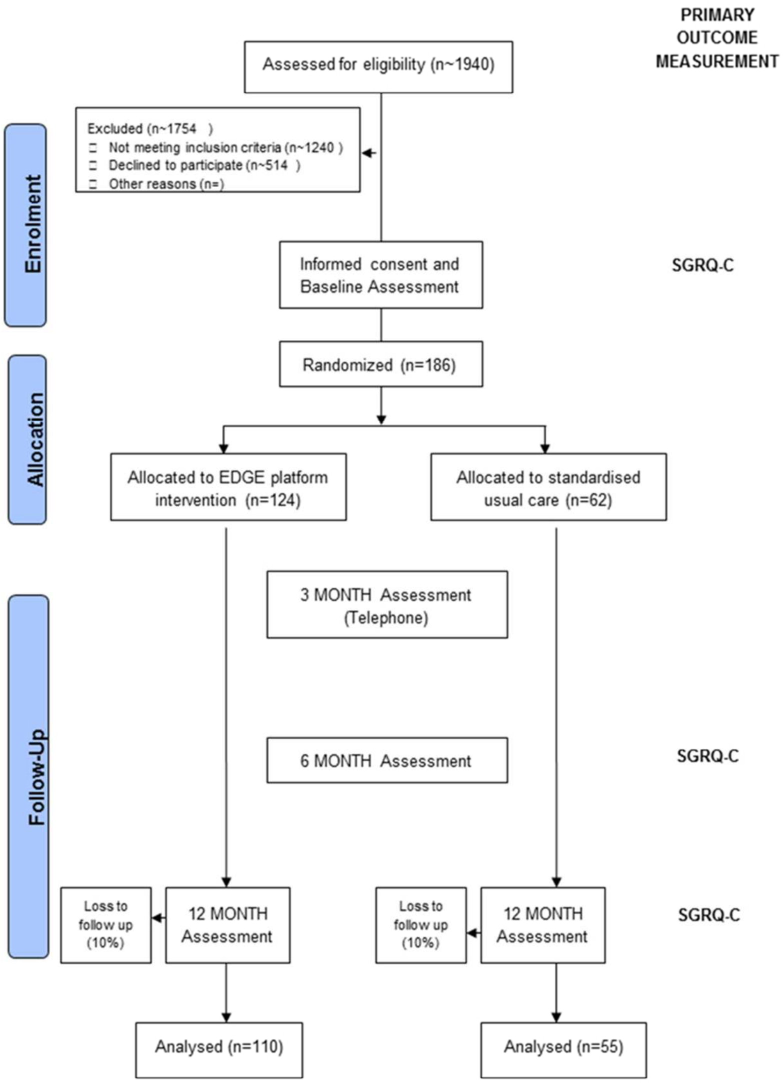


Figure 1 - Patient Flow
142x190mm (150 x 150 DPI)

| | Baseline assessment visit | Three months | Six months | Twelve months |
|---|---|---|---|---|
| Primary outcome | | | | |
| SGRQ-C (quality of life)[18] | x | | x | x |
| Secondary outcomes | | | | |
| Number of exacerbations | x | x | x | x |
| Time to first exacerbation (days) | | x | x | x |
| Unplanned healthcare contacts | | x | x | x |
| EuroQol 5 Dimension Questionnaire[22] | x | | x | x |
| Self-Completed 20 item depression measure D[20] | x | | x | x |
| Self-Completed 10 item anxiety measure [21] | x | | x | x |
| Beliefs about medicines questionnaire[19] | x | | x | x |
| Medication adherence report schedule[19] | x | | x | x |
| Prescribed medicines | x | x | x | x |
| Smoking status | x | | x | x |
| Health Service usage | x | x | x | x |
| Death | | x | x | x |
| Admission to hospital | x | x | x | x |
| Days out hospital | x | x | x | x |

Table 1 - Outcome measures matrix
158x157mm (96 x 96 DPI)

nly

# Self-management support using an internet-linked tablet computer (the EDGE platform) based intervention in Chronic Obstructive Pulmonary Disease: protocol for the EDGE-COPD randomized controlled trial.

Andrew Farmer[1*], Christy Toms[1], Maxine Hardinge[2], Veronika Williams[1], Heather Rutter[3], Lionel Tarassenko[4].

* Corresponding author
23-38 Hythe Bridge Street, Oxford OX1 2ET
andrew.farmer@phc.ox.ac.uk tel: 01865 289300 fax: 01865 289412

1. Department of Primary Care Health Sciences, University of Oxford, Oxford, UK
2. Oxford University Hospitals NHS Trust, Oxford, UK
3. Oxford Health NHS Foundation Trust, Oxford, UK
4. Institute of Biomedical Engineering, University of Oxford, Oxford, UK

**Keywords:** COPD, Telemedicine, primary care, respiratory medicine.

**Word Count:** ~~3642~~3892



For peer review only - http://bmjopen.bmj.com/site/about/guidelines.xhtml

## ABSTRACT

**Introduction:** The potential for telehealth-based interventions to provide remote support, education and improve self-management for long term conditions is increasingly recognised. This trial aims to determine whether an intervention delivered through an easy-to-use tablet computer can improve the quality of life of patients with chronic obstructive pulmonary disease (COPD) by providing personalised self-management information and education.

**Methods and analysis:** The sElf management anD support proGrammE (EDGE) for COPD is a multi-centre, randomised controlled trial designed to assess the efficacy of an internet-linked tablet computer based intervention (the EDGE platform) in improving quality of life in patients with moderate to very severe COPD compared with usual care. Eligible patients are randomly allocated to receive the tablet computer based intervention or usual care in a 2:1 ratio using a web-based randomisation system. Participants are recruited from respiratory outpatient clinics and pulmonary rehabilitation courses as well as from those recently discharged from hospital with a COPD-related admission and from primary care clinics. Participants allocated to the tablet computer based intervention complete a daily symptom diary and record clinical symptoms using a Bluetooth-linked pulse oximeter. Participants allocated to receive usual care are provided with all the information given to those allocated to the intervention but without the use of the tablet computer or the facility to monitor their symptoms or physiological variables. The primary outcome of quality of life is measured using the St George's respiratory questionnaire for COPD patients (SGRQ-C) baseline, 6 and 12 months. Secondary outcome measures are recorded at these intervals in addition to 3 months.

**Ethics and dissemination:** The Research Ethics Committee for Berkshire – South Central has provided ethical approval for the conduct of the study in the recruiting regions. The results of the study will be disseminated through peer review publications and conference presentations.

**Trial registration:** Current controlled trials ISRCTN40367841




For peer review only - http://bmjopen.bmj.com/site/about/guidelines.xhtml

## Article Summary

### Article focus

- This protocol paper describes a randomised controlled trial of a novel implementation of telehealth in improving quality of life in patients with Chronic Obstructive Pulmonary Disease.

### Key messages

- The study aims to explore the effectiveness of a mobile, tablet computer based intervention incorporating an individualised self management package in improving patients' quality of life.
- The intervention has been specifically designed to be easily incorporated into day-to-day life and clinical care management.

### Strengths and limitation of this study

- The study is powered to examine quality of life outcomes and uses an unbalanced allocation to examine the effect of the EDGE platform across a wide range of participants.
- The study is not sufficiently large enough to provide a detailed cost-effectiveness evaluation, or to provide sufficient power to demonstrate clinically important differences between intervention and usual care groups for hospital admission rates.



For peer review only

For peer review only - http://bmjopen.bmj.com/site/about/guidelines.xhtml

## BACKGROUND

Chronic obstructive pulmonary disease (COPD) is an important cause of morbidity and mortality worldwide responsible for three million deaths globally per year. [1] In the UK the total annual estimated cost of COPD to the NHS is over £800 million, with over half of this attributable to hospital-based care,[2] and the impact of COPD to the health-related quality of life of patients is well established. [3, 4] There is now promising evidence that training and support for patients in the self management of their condition improves quality of life and can reduce unplanned hospital admissions. [5,6] However, the results of individual studies are mixed, [7] and the challenge remains to identify those for whom different forms of self-management are suitable and to develop and optimise ways of delivering available interventions to maximise effectiveness and safety.

Use of the converging computer and communication technologies in the form of telehealth-based interventions offers a means of helping patients monitor their condition, providing support in interpreting data, providing a means of delivering individually tailored education and treatment plans, and allowing clinicians to monitor long-term trends and identify short term safety issues.

Evaluation of telehealth-based interventions can be complex with a requirement for considering multiple perspectives within the evaluation. [8] For a successful evaluation there is a need to improve the delivery of clinical care, control the workload placed on healthcare professionals, and to provide cost effective improvements in treatment outcomes.

Systematic reviews of telehealth in COPD provide evidence to support continuing research, [9] but recent large-scale evaluations of telehealth-based treatment programmes have not shown convincing evidence of effectiveness. [10,11] The challenge remains to ensure that the telehealth systems can be easily used by patients and that the systems integrated into the health care system are acceptable to clinicians and patients . Criticism of previous systems has included difficulty with data entry, leading to lifestyle restriction, unreliability of monitoring devices, and lack of integration within an individual patient's day-to day life.

Our group has long experience of developing and testing health care interventions based on the use of mobile phones and their integration into clinical care delivery. Mobile phones or tablet computers with a Subscriber Identity Module (SIM) card provide a platform in which communication and computing technologies are sustainably integrated, and provide a platform for communicating with monitoring devices. We have recently carried out a cohort



For peer review only - http://bmjopen.bmj.com/site/about/guidelines.xhtml

study in which we have shown that a tablet-computer based system for supporting patients with COPD is acceptable and feasible.[12]

We have therefore set out to determine the efficacy of an internet-linked tablet computer based intervention (the EDGE - sElf management anD support proGrammE - platform), with patients with moderate to very severe COPD, in improving quality of life measured with the St Georges Respiratory questionnaire for COPD patients (SGRQ-C) in comparison with standardised usual care. In addition we will collect data on morbidity, mortality and hospital admissions to inform the design of future evaluations of the system.

## METHODS

### Trial Design

The sElf management anD support proGrammE (EDGE) for COPD is a multi-centre, randomised controlled trial of twelve-month duration. Patients are individually randomised to receive either an internet-linked tablet computer (the EDGE platform) based intervention or standardised usual care in a 2:1 allocation ratio (Figure 1).

### Participants

*Eligibility criteria for participants*

Eligible patients are those aged ≥40 years of age with a confirmed diagnosis of chronic obstructive pulmonary disease (COPD) defined as a forced one second expiratory volume (FEV1) post-bronchodilation of <80% and a predicted ratio of FEV1 to forced vital capacity (FVC) of <0.70. Eligible patients have a smoking pack history >10 pack years and an MRC dyspnoea score ≥ 2.A clinical decision of trial suitability for patients who are unable, for clinical reasons, to provide a spirometry reading at full assessment is sufficient for eligibility if the patient has prior clinical evidence of COPD (e.g. obstructive spirometry within the last 10 years or radiological evidence of emphysema). Patients are required to be registered with a general practitioner and have had an exacerbation of COPD requiring home treatment or hospital admission in the previous year or have been referred for pulmonary rehabilitation.

Patients are not eligible for trial participation if they have ~~a smoking history of <10 pack years, an MRC dyspnoea score <2,~~ other significant lung disease or chronic heart failure



For peer review only - http://bmjopen.bmj.com/site/about/guidelines.xhtml

(defined by the New York Heart Association classification system as severe (grade IV)) or a life expectancy of <3 months. Eligible patients must be able to provide informed consent, to complete the trial questionnaires and not be cognitively impaired. Patients living in areas without access to a mobile phone network able to provide access to the Internet, and thus unable to transmit and receive data are not eligible to enter the trial.

*Setting*

To maximise recruitment of participants to the trial, patients are identified from a variety of settings encompassing primary and secondary care as well as community services. ~~Participants are recruited from patients~~Patients attending respiratory hospital outpatient clinics and pulmonary rehabilitation courses in the adjacent counties of Oxfordshire and Berkshire, United Kingdom (UK) are invited to participate. In addition, eligible patients are identified from primary care clinics and from those recently (within the preceding two weeks) discharged from hospital following a COPD-related admission.

**Trial interventions**

*Intervention development and specification*

We developed and tested an intervention to support patients with COPD in monitoring their health and to provide information and education about their condition based on use of an internet-linked tablet computer (the EDGE platform). Based on open architecture application software, the EDGE platform was developed to allow integration within clinical care by a team of clinicians and engineers working with patients. The platform was refined in a six-month cohort study with a group of COPD patients selected using eligibility criteria matching those of the trial. Key principles underlying the development of the platform include ease of use for a group of participants less experienced with computers (large icons, no keyboard needed for data entry) and ensuring data quality (tablet based instructions to ensure ease of use, development of algorithms to assure data quality).

The EDGE platform intervention incorporates a daily symptom diary consisting of a series of standard questions about symptoms based on previous trial protocols. [2,13,14] Questions include general well-being, cough, breathlessness, sputum (quantity produced and colour) and use of medications. A thirty-second period of data transmission using a Bluetooth-enabled pulse oximeter with finger probe allows daily collection of pulse rate and oxygen saturation data. Every four weeks, beginning two weeks after initial use, the platform presents



For peer review only - http://bmjopen.bmj.com/site/about/guidelines.xhtml

the four-item Patient Health Questionnaire (PHQ-4) screening measure[5,15] and if either the depression or anxiety components score is ≥3 then the relevant full questionnaire (depression (PHQ-8,[6,16]) or anxiety (GAD7,[7,17]) is presented for completion.

The EDGE platform also includes software modules to provide patients with additional support for self-management of their condition. These include (i) personalised plans for self-management and treating an exacerbation of their condition (ii) brief-video clips and text-based material providing additional information about COPD and treatments (including medicines use and inhaler technique), and educational advice on managing COPD, smoking cessation, diet, physical activity, and mood management (depression and anxiety), and (iii) the facility to receive a brief message from their respiratory nurse. Prompts for accessing the physical activity and mood management information are included for patients reporting higher scores on the depression and anxiety screening measures.

The data entered onto the EDGE platform is transmitted immediately to a server hosted behind NHS firewalls. The data held on the server is reviewed at no less than four-day intervals by a clinician to identify technical problems and review clinical data received.

In an initial six-week period of use, EDGE-platform users complete the symptom diary and record their oxygen saturation and heart rate with the pulse oximeter on a daily basis. Following this initial run-in period of use, the distributions of values for the oxygen saturation, heart rate and symptom scores are calculated for the run-in period for each participant. The 95$^{th}$ centile is computed for each distribution and used as the threshold for the participant's safety alert for that parameter. Every time the participant-specific threshold for a parameter is crossed after the run-in period, this safety alert is displayed on the web-based record for that participant.

*The EDGE platform based intervention*

Participants allocated to receive the Internet-linked tablet computer (EDGE platform)-based intervention are provided with an Android tablet computer (Samsung Galaxy Tab) running the application software and Bluetooth-enabled oximeter probe (Nonin, PureSAT, 956OBT, Nonin Medical Inc, Plymouth Minnesota). Participants are briefly instructed on the use of the EDGE platform by the research nurse and are provided with a brief information booklet giving details of its use. Participants continue to input their symptom data and clinical recordings daily throughout the duration of the trial. Patients are informed that the EDGE system is not a replacement for their usual clinical care, and that in the event of deterioration



For peer review only - http://bmjopen.bmj.com/site/about/guidelines.xhtml

in their health they should contact their general practitioner or community respiratory nurse as usual. However, data are reviewed by a clinician at no less than four-day intervals, and changes in heart rate, oxygen saturation or symptom score that cross the participant's specific threshold for that parameter and persist for more than four days are followed up with a phone call to either the patient, general practitioner or community respiratory nurse as appropriate. If depression or anxiety scores equal or exceed a threshold of 10, then the general practitioner is informed by letter.

*The standardised usual care intervention*

Participants allocated to receive standardised usual care are provided with all the information given to those allocated to use the EDGE platform, but without the use of a tablet computer or the facility for daily monitoring of symptoms and physiological variables. The research nurse provides participants with leaflets based on those currently produced by the Oxfordshire Community Respiratory service. Personalised information intended to help patients understand their condition includes information about how to use their medications and when they should be used, a self-management plan with written guidelines on what to do and who to contact if they experience an exacerbation and dietary advice is provided. If the participant has not attended a pulmonary rehabilitation course they are invited to do so.

**Outcomes**

Trial outcome measures will be recorded as indicated in Table 1.

*Primary outcome measure*

The primary outcome is the St George's Respiratory Questionnaire for COPD patients (SGRQ-C). [8,18] used to assess quality of life in patients with moderate to severe COPD.

*Secondary outcome measures*

The following secondary endpoints will be used to evaluate the impact of the intervention in comparison to usual care (i) impact on hospital admissions (number of admissions and days out of hospital) and deaths; (ii), the number of recorded exacerbations defined as episodes in which antibiotics or oral steroids were prescribed or in which the patients were seen in the Accident and Emergency Department and/or admitted to hospital as a result of a respiratory episode; (iii) time to first exacerbation; (iv) beliefs about respiratory medicine use measured with the Beliefs about Medicines Questionnaire; [9,19] (v) self-reported medication use



For peer review only

measured with the Medication Adherence Report Schedule; [10,11,19] (vi) self-reported smoking cessation; (vii) mood measured with the standard checklist 20-item questionnaire (SCL-20) for depression[20] and the standard checklist 10-item anxiety measure (SCL-10A); [21] and (viii) health status measured with the EuroQol 5-Dimension Questionnaire (EQ-5D). [22]

Details of number and duration of hospital admissions will be measured by self-report and confirmed where possible by a review of hospital discharge letters and central hospital admissions data. Records of deaths will be obtained from general practices and further details obtained where necessary from hospital records.

Details of exacerbations of COPD will be recorded on a record form held by all participants. This will include number, severity, medications prescribed and outcomes to enable, where possible, details to be confirmed by review of medical records or, if deterioration results in admission, review of hospital records.

Costs of healthcare will be identified using a brief questionnaire to obtain self-reported information about visits to general practitioners. Standard costs will be used for hospital admissions and prescribed respiratory medicine.

**Sample size**

The sample size calculations are based on the numbers of patients required to demonstrate a mean difference of 6.6 on the St George's Respiratory Questionnaire between the two trial groups to which participants are allocated, over a twelve-month period (equivalent to 7.3 on SGRC-C).[18] Although there is limited data on this outcome in settings using computer-based interventions, we have estimated the standard deviation at 12.7 based on a study using the SGRC. [23] A trial using these estimates with a power of 90% and significance level of 0.05 (2-sided), with 2:1 allocation between intervention and usual care and allowing for 10% loss to follow-up would require 165 patients.

We also have 98% power to identify a difference in admissions to hospital at 3-months based on effect sizes of previous intensive interventions with this group of patients,[24] and 52% power to detect a difference in admissions at twelve months based on a systematic review of interventions in COPD.[25] In both cases a 5% loss to follow-up has been assumed.

**Randomisation**

Participants are randomised with an allocation ratio of 2:1 intervention to usual care using Sortition V1.2 (an online, web-based randomisation system developed by the Primary Care



For peer review only - http://bmjopen.bmj.com/site/about/guidelines.xhtml

Clinical Trials Unit at the University of Oxford). A computer schedule based on recruiting site (Oxfordshire or Berkshire), age (≤70 years or >70 years), gender, COPD severity (moderate or severe/very severe) and current smoking status (yes or no) is used to minimise imbalance between the groups,[26] and is monitored by an independent statistician. The research nurse randomises the patient by accessing Sortition using a web-browser on a tablet computer at the assessment visit only after completion of consent procedures and baseline measurements, including completion of the SGRC-C.

Self-completed outcome measures at six and twelve months are completed without guidance by the research team and prior to any further assessment or discussion of clinical care. Research and clinical teams are trained in the potential for measures to be biased by their interactions with participants. A record of all contacts with trial participants is kept to examine potential for interactions with patients not specified in the trial protocol.

**Trial Procedures**

*Recruitment*

Potentially eligible patients are identified from those discharged from hospital following a COPD-related admission, from respiratory hospital outpatient clinics, pulmonary rehabilitation courses and from primary care clinics, and are sent an invitation to participate in the trial. The invitation includes a patient information booklet, a reply slip and pre-paid envelope. Patients interested in participating are asked to return their reply slips by post to the research team. The research nurse then contacts the patient by telephone to arrange an initial assessment visit. At this visit eligibility is confirmed, written informed consent obtained and baseline data collected for those consenting to participate.

All participants are assessed at baseline by a ~~clinician~~ healthcare professional and complete self-completed measures prior to randomisation and intervention allocation. The use of medication by participants is recorded at the baseline and follow-up assessment visits. Information collected includes type, dose and frequency of COPD medication (tablets, inhalers) as well as a list of other medication taken. A detailed smoking history is taken at the baseline assessment visit; self-reported smoking status is recorded at subsequent assessments.

*Patient follow-up and retention*

**Formatted:** Font: Italic

Patients remain in the trial for 12 months with assessments at a baseline visit, three, six and twelve months. The primary outcome measure is collected at baseline, and six and twelve



For peer review only - http://bmjopen.bmj.com/site/about/guidelines.xhtml

months after randomisation. Secondary outcome measures are collected at baseline, three, six and twelve months.

The three-month visit assessment is a telephone contact with patients sent reminders prior to the assessment date. For patients allocated to standardised usual care a reminder is posted prior to the assessment date. For patients receiving the EDGE platform intervention, this reminder is sent in the form of a message to the tablet computer. , The six and twelve month visits are carried out either at home or at clinic, with patients receiving postal or electronic reminders prior to the assessment visit date. The researcher ensures at each visit that all outcome measures are complete to ensure maximal follow-up data is collected. the six and twelve-month visits are carried out either at home or at clinic whichever is more convenient for the participant.

All patients have the right to withdraw from the trial at any point, without providing a reason. Those patients who do withdraw from the trial will be asked if they would be willing to provide follow-up information at the 6 and 12 month assessment points. If patients decline no further information will be collected.

**Statistical methods**

The principal comparisons will be performed on an intention-to-treat basis. The trial results will be presented as comparative summary statistics (difference in response rates or means) with 95% confidence intervals. If appropriate, and depending on the distribution of the continuous outcome measures, a linear mixed-effects model will be used to analyse SGRQ-C over the twelve month period of the trial, adjusting for baseline value and minimisation covariates. Treatment-time interaction will be included in the model to assess the treatment effect at twelve months. We will formally assess the distribution of the change from baseline for evidence of departure from normality. If necessary, data will either be transformed or analysed using a non-parametric equivalent. The nature and mechanism for the missing outcomes will be investigated, though mixed effects models implicitly account for data missing at random. Sensitivity analyses will be carried out to examine the robustness of the results with different assumptions about departures from randomisation policies, and handling of missing data. Binary outcomes will be analysed using log-binomial regression, adjusting for covariates as described above.

The intervention effect will be assessed by analysis of sub-groups defined by severity of COPD, smoking status, hospital admission in the previous year, attendance on a pulmonary



For peer review only - http://bmjopen.bmj.com/site/about/guidelines.xhtml

rehabilitation course in the previous year and the presence or absence of live-in support. ~~A~~ The full detailed statistical analysis plan, including any pre-specified subgroup and sensitivity analyses, will be prepared before the final analysis by the trial statistician.

**Embedded qualitative process evaluation**

An embedded qualitative study will involve individual interviews with a sub group of up to 30 patients in the intervention group invited to take part. Interviews will take place at baseline (after patients have been randomised and prior to the delivery of the EDGE platform based intervention) and after the 12-month visit, and will be carried out by a qualitative researcher. The aim of the baseline interview is to explore patients' current self-management strategies, whilst the second interview will focus on how the EDGE platform intervention has impacted on their self-management of COPD, and explore issues of acceptability, everyday use and usability.

A maximum variation sample in terms of age, gender, employment status, care support, severity of COPD, exacerbation frequency and experience of using computer or smart phone technology will be sought. Interviews will be audio-recorded and transcribed verbatim. NVivo© will be used to facilitate organisation and analysis of data. Analytical procedures will follow grounded theory methods, [27] including double coding by a second qualitative researcher to ensure rigour.

**Ethical approvals**

The EDGE COPD trial will be carried out in conformance with the principles of the current version of the Declaration of Helsinki and the other regulations in force. Ethical approval has been received from the South Central - Berkshire Research Ethics Committee of the United Kingdom National Research Ethics Service (Ethics Ref: 12/SC/0437). The trial analysis and reporting will be independent of the trial funder and sponsor. The trial is registered with Current Controlled Trials ISRCTN40367841.

## DISCUSSION

This trial is designed to assess the extent to which a telehealth platform designed to support self-management improves quality of life for people with chronic obstructive pulmonary disease. Reduced quality of life is an important aspect of COPD that has been shown to be



For peer review only

responsive to group and one-to-one interventions. [7] This trial is powered to show a clinically important effect, and provide data from which to design further trials to explore cost-effectiveness and potentially reduction in hospital admissions.

The trial intervention uses a novel implementation of telehealth using a non-proprietary tablet computer designed to be integrated into day-to-day life and clinical care. In addition to being non-obtrusive it provides, at relatively low cost in relation to previous telehealth systems, facilities for monitoring, communication, self-management support and education delivery. The development of the system was carried out iteratively using best practice to involve patients, engineers and clinicians in repeated testing and assessment. [28]

The underlying approach to implementing telehealth within this trial is to provide a system focussed around the needs of the patient with collection of data that can be analysed over a period of time and used to inform future management. Although future implementations of this system may allow real-time alerts to inform management, consistent experience to date with existing commercial telehealth systems has been that the high rates of false-positive alerts have been unacceptable to clinicians and patients. This trial will provide data to evaluate the potential of the patient-specific tailored alerts available in the system for implementation in practice.

The current evaluation has a number of strengths in comparison with previous trials of telehealth systems. In particular the information provided by the platform is timely, and immediately available, but does not provide an overwhelming flow of data. The platform has been evaluated for acceptability and feasibility in a cohort study over a period of six months in which the tablets were used daily by over 80% of participants. The process of completing diaries and measuring oxygen saturation takes less than three minutes, with an average of five video segments viewed by each participant during their six-month participation in the cohort study. A qualitative process evaluation adds to the strength of the design. The unbalanced allocation will provide additional power to examine the effect of the EDGE platform across a wide range of participants. The trial however, is not sufficiently large to provide a detailed cost-effectiveness evaluation, nor to provide sufficient power to demonstrate clinically important differences between intervention and usual care groups for hospital admission rates. The trial is due to report in September 2015.



For peer review only - http://bmjopen.bmj.com/site/about/guidelines.xhtml

## ACKNOWLEDGEMENTS

The authors would like to acknowledge the EDGE COPD Trial group consisting of, in addition to the authors of this manuscript, Dr Jonathan Price, Dr Carl Heneghan and Dr Gari Clifford who provided clinical and scientific input into the design of the intervention and trial and the trial engineering team including Dr Oliver Gibson, Dr Carmelo Velardo and Dr Syed Ahmar Shah who developed and delivered the EDGE platform. Grace Robinson (respiratory consultant and principal investigator), Katherine Thomas (respiratory clinician) and Jacqui Webb (research nurse) lead recruitment at the Royal Berkshire Hospital Department of Respiratory Medicine.

## FOOTNOTES

**Contributors:** LT and AF conceived the original idea for the trial and all authors were involved in the development of the intervention and design of the trial. AF, assisted by CT drafted the initial manuscript. All authors commented on subsequent drafts and approved the final version.

**Funding:**

This ~~work was supported by the Wellcome Trust and Department of Health, Health Innovation Challenge Fund; grant number HICF-1010-032.~~project is funded through a Health Innovation Challenge Fund award [HICF-1010-032] provided by the Wellcome Trust and Department of Health. The trial is sponsored by the University of Oxford. Neither organisation has been involved in the design of the study, the collection, management, analysis and interpretation of data; writing of the report; and the decision to publish trial results. Oversight for these activities resides with the EDGE COPD Trial group.

LT and AF receive funding from the Oxford NIHR BRC. AF is an NIHR Senior Investigator.

**Competing interests:**

None



For peer review only

For peer review only - http://bmjopen.bmj.com/site/about/guidelines.xhtml

## REFERENCES


1 World Health Organization. *The global burden of disease: 2004 update*. Geneva: : WHO 2008.

2 National Clinical Guideline Centre. *Chronic obstructive pulmonary disease: Management of chronic obstructive pulmonary disease in adults in primary and secondary care*. London: : NICE 2010.

3 Okubadejo AA, O'Shea L, Jones PW, *et al.* Home assessment of activities of daily living in patients with severe chronic obstructive pulmonary disease on long-term oxygen therapy. *European Respiratory Journal* 1997;**10**:1572–5.

4 Ståhl E, Lindberg A, Jansson S-A, *et al.* Health-related quality of life is related to COPD disease severity. *HealthQualLifeOutcomes* 2005;**3**:56–6.

5 Bourbeau J, Julien M, Maltais F, *et al.* Reduction of hospital utilization in patients with chronic obstructive pulmonary disease: a disease-specific self-management intervention. *ArchInternMed* 2003;**163**:585–91.

6 Rea HH, McAuley SS, Stewart AA, *et al.* A chronic disease management programme can reduce days in hospital for patients with chronic obstructive pulmonary disease. *Intern Med J* 2004;**34**:608–14.

7 Effing T, Monninkhof EM, van der Valk PDLPM, *et al.* Self-management education for patients with chronic obstructive pulmonary disease. *Cochrane Database SystRev* 2007;:CD002990.




For peer review only - http://bmjopen.bmj.com/site/about/guidelines.xhtml

8 Greenhalgh T, Procter R, Wherton J, *et al.* The organising vision for telehealth and telecare: discourse analysis. *BMJ Open* 2012;**2**:e001574–4.

9 Polisena J, Tran K, Cimon K, *et al.* Home telehealth for chronic obstructive pulmonary disease: a systematic review and meta-analysis. *JTelemedTelecare* 2010;**16**:120–7.

10 Steventon A, Bardsley M, Billings J, *et al.* Effect of telehealth on use of secondary care and mortality: findings from the Whole System Demonstrator cluster randomised trial. *BMJ* 2012;**344**:e3874–4.

11 Fairbrother PP, Pinnock HH, Hanley JJ, *et al.* Continuity, but at what cost? The impact of telemonitoring  COPD on continuities of care: a qualitative study. *Prim Care Respir J* 2012;**21**:322–8.

12 M-Health for long term management of COPD. The Kings Fund, London:  2013.

13 Time course and recovery of exacerbations in patients with chronic obstructive pulmonary disease. 2000;**161**:1608–13.

14 Bucknall CE, Miller G, Lloyd SM, *et al.* Glasgow supported self-management trial (GSuST) for patients with moderate to severe COPD: randomised controlled trial. *BMJ* 2012;**344**:e1060–0.

15 A 4-item measure of depression and anxiety: Validation and standardization of the Patient Health Questionnaire-4 (PHQ-4) in the general population. 2010;**122**:86–95.

16 Kroenke K, Strine TW, Spitzer RL, *et al.* The PHQ-8 as a measure of current depression in the general population. *Journal of Affective Disorders* 2009;**114**:163–73.

17 A brief measure for assessing generalized anxiety disorder: the GAD-7. 2006;**166**:1092–7.

18 Meguro M, Barley EA, Spencer S, *et al.* Development and Validation of an Improved, COPD-Specific Version of the St. George Respiratory Questionnaire. *Chest* 2007;**132**:456–63.

19 Horne R, Weinman J, Hankins M. The beliefs about medicines questionnaire: The development and evaluation of a new method for assessing the cognitive representation of medication. *PsycholHealth* 1999;**14**:1–24.

20 Derogatis LRL, Lipman RSR, Covi LL. SCL-90: an outpatient psychiatric rating scale--preliminary report. *Psychopharmacol Bull* 1973;**9**:13–28.

21 The Hopkins Symptom Checklist (HSCL): a self-report symptom inventory. 1974;**19**:1–15.

22 Group E. EuroQol: a new facility for the measurement of health related quality of life. *Health Pol* 2001;**16**:199–208.

23 Welte T, Miravitlles M, Hernandez P, *et al.* Efficacy and Tolerability of Budesonide/Formoterol Added to Tiotropium in Patients with Chronic Obstructive Pulmonary Disease. *Am J Respir Crit Care Med* 2009;**180**:741–50.

24 Seymour JM, Moore L, Jolley CJ, *et al.* Outpatient pulmonary rehabilitation following acute exacerbations of COPD. *Thorax* 2010;**65**:423–8.

25 McLean S, Nurmatov U, Liu JL, *et al.* Telehealthcare for chronic obstructive pulmonary disease. *Cochrane Database SystRev* 2011;:CD007718.

26 Altman DG, Bland JM. Treatment allocation by minimisation. *BMJ* 2005;**330**:843.



For peer review only

For peer review only - http://bmjopen.bmj.com/site/about/guidelines.xhtml

27 Charmaz K, Mitchell RG. Grounded theory in Ethnography. In: Atkinson P, Coffey A, Delamont S, *et al.*, eds. *Handbook of ethnography*. London: : SAGE Publications 2001.

28 Campbell NC, Murray E, Darbyshire J, *et al.* Designing and evaluating complex interventions to improve health care. *BMJ* 2007;**334**:455–9.

For peer review only



For peer review only - http://bmjopen.bmj.com/site/about/guidelines.xhtml
